# Large-scale investigation of the reasons why potentially important genes are ignored

**DOI:** 10.1371/journal.pbio.2006643

**Published:** 2018-09-18

**Authors:** Thomas Stoeger, Martin Gerlach, Richard I. Morimoto, Luís A. Nunes Amaral

**Affiliations:** 1 Center for Genetic Medicine, Northwestern University, Chicago, United States of America; 2 Northwestern Institute on Complex Systems (NICO), Northwestern University, Evanston, United States of America; 3 Department of Chemical and Biological Engineering, Northwestern University, Evanston, United States of America; 4 Department of Molecular Bioscience, Northwestern University, Evanston, United States of America; 5 Department of Physics and Astronomy, Northwestern University, Evanston, United States of America; University of Edinburgh, United Kingdom of Great Britain and Northern Ireland

## Abstract

Biomedical research has been previously reported to primarily focus on a minority of all known genes. Here, we demonstrate that these differences in attention can be explained, to a large extent, exclusively from a small set of identifiable chemical, physical, and biological properties of genes. Together with knowledge about homologous genes from model organisms, these features allow us to accurately predict the number of publications on individual human genes, the year of their first report, the levels of funding awarded by the National Institutes of Health (NIH), and the development of drugs against disease-associated genes. By explicitly identifying the reasons for gene-specific bias and performing a meta-analysis of existing computational and experimental knowledge bases, we describe gene-specific strategies for the identification of important but hitherto ignored genes that can open novel directions for future investigation.

## Author summary

Biomedical research is one of the largest areas of present-day science and embeds the hope and potential to improve the lives of the general public. In order to understand how individual scientists choose individual research questions, we study why certain genes are well studied but others are not. While it has been previously observed that most research on human genes only concentrates on approximately 2,000 of the 19,000 genes of the human genome, the reasons for this ignorance are largely unknown. We systematically test explanations for this observation by compiling an extensive resource that characterizes biomedical research, including but not limited to hundreds of chemical and biological properties of gene-encoded proteins, and the published scientific literature on individual genes. Using machine learning methods, we can predict the number of publications on individual genes, the year of the first publication about them, the extent of funding by the National Institutes of Health, and the existence of related medical drugs. We find that biomedical research is primarily guided by a handful of generic chemical and biological characteristics of genes, which facilitated experimentation during the 1980s and 1990s, rather than the physiological importance of individual genes or their relevance to human disease.

## Introduction

Recent studies have demonstrated the highly imbalanced research effort directed towards individual human protein-coding genes [1–8], which manifests itself in several ways, including the number of publications per gene, the number of human-curated and computationally predicted functional annotations, the number of gene names and gene symbols, and the number of patents containing their nucleotide sequences (S1 Fig). Plausibly, this observed disparity could reflect a lack of importance of many genes, but more likely it could also reflect existing social structures of research [9, 10], scientific and economic reward systems [11, 12], medical and societal relevance [13–15], preceding discoveries [2, 16], serendipity [17, 18], the availability of technologies [19, 20] and reagents [6, 21], and other intrinsic characteristics of genes [22–24]. It remains unclear, however, if any of these factors can significantly explain the observed number of publications on individual human genes. Nor is it known whether descriptions about the formation of scientific knowledge translate into gene-specific insight, and whether these reasons for historically grown bias could already be mitigated by current experimental possibilities.

In order to address these challenges, we created a database cross-referencing chemical, physical, biological, historical, bibliometric, financial, technological, and experimental data on all human protein-coding genes from 36 different sources (see Materials and methods). Using this resource, we show how characteristics of genes relate to the macroscopic output of biomedical research in terms of the number of publications, perceived biological importance of genes, funding, and translational activities. We show different examples of how this resource can be used to define strategies for a more efficient exploration of the space of biological functions, and provide high-level gene-specific analyses in a series of supplementary tables.

## Results

### Intrinsic gene characteristics suffice to predict publications

**Fig 1:**
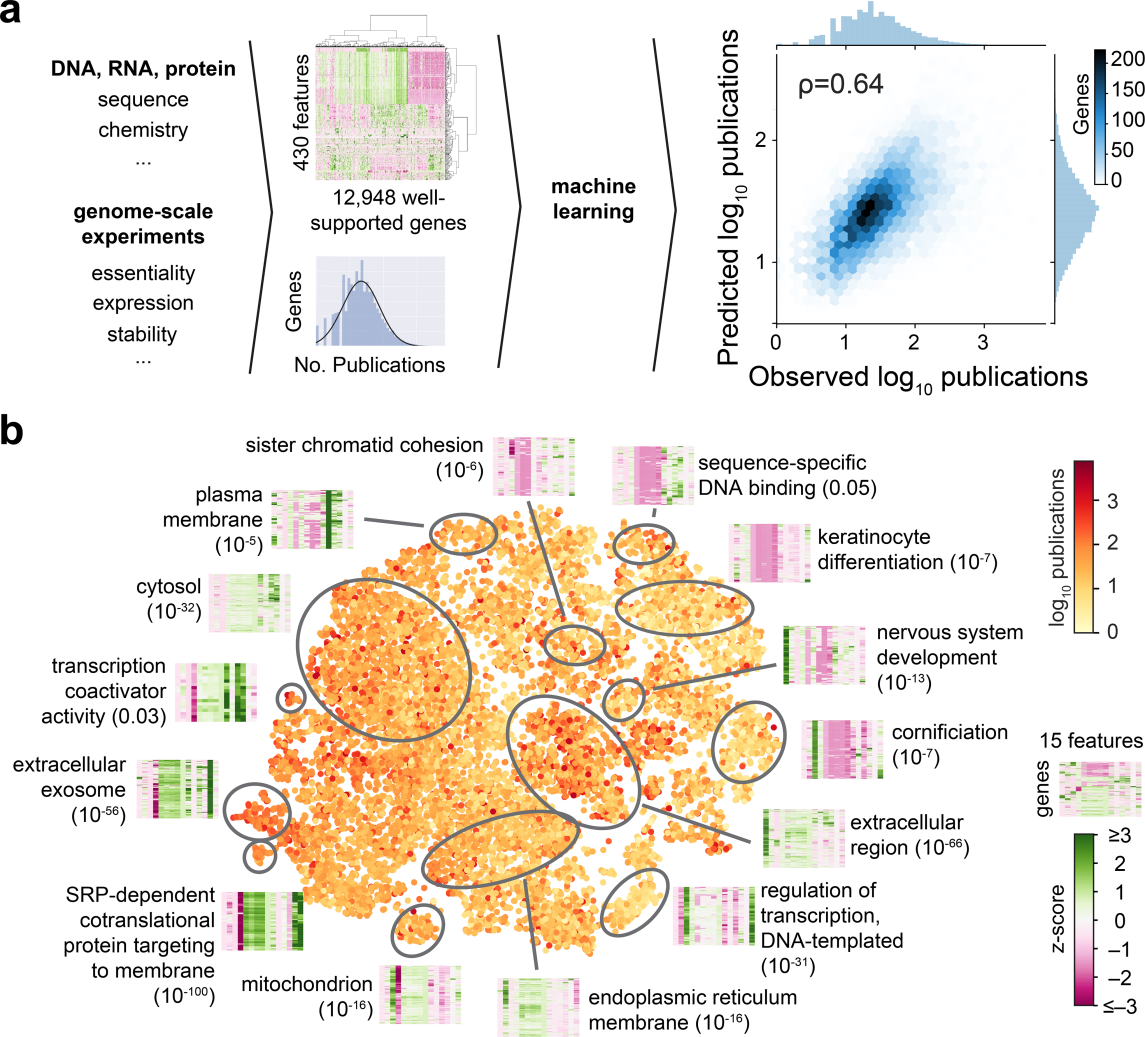
Physical, chemical, and biological features of genes predict the number of publications. **(A)** Illustration of modeling approach and prediction of number of research publications for single genes using information on 430 physical, chemical, and biological features of genes (S1 Data). **(B)** Research publications on individual genes grouped by t-SNE visualization using the 15 features most important to the models used in **(A)**. Heatmaps show z-scored values of the 15 features for the genes in each cluster. Order of features as indicated in S3A Fig (S1 Data). SRP, signal recognition particle; t-SNE, t-distributed stochastic neighbor embedding.

To test if measurable intrinsic chemical, physical, and biological features of genes and gene products alone suffice to describe the number of publications per gene, we gathered 430 features per gene, which could either be computed from known sequences of these genes or obtained from previously published genome-scale experiments (Fig 1A). Intriguingly, we observed that 33% of the protein-coding genes carrying an official gene name had an incomplete catalog of features. The dominant reasons for the absence of features were the absence of reported insertions within recent Clustered Regularly Interspaced Short Palindromic Repeats (CRISPR) loss-of-function screens (about 13% of genes, depending on assay), the absence of detectable RNA across all tissues and cell lines surveyed by the human protein atlas (6% of genes), the absence of validated RNA molecules within the Genbank reference database of RNA molecules (5% of genes), and the absence of reported protein molecules within the UniProt reference database for protein molecules (3% of genes) (S2A Fig, S1 Table). Foreshadowing our subsequent analyses, the absence of reported features correlated with a lower number of reported publications (S2A Fig). This initial result illustrates limitations in experimental approaches and a surprising degree of uncertainty that remains about human genes and the existence of their gene products.

To assess whether the values of these features, rather than solely their presence, would quantitatively inform on the number of publications of individual genes, we proceeded by only considering the 12,948 genes with a complete set of features (S2 Table). Using gradient boosting regressions with out-of-sample Monte Carlo cross-validation [25], we could predict to a significant extent the number of publications on any given gene (Fig 1A, Spearman: 0.64). Remarkably, 15 out of 430 features contributed the most to our model’s accuracy (S3A Fig) and fell into six categories that specify the abundance of gene-encoded RNA and protein molecules across multiple tissues (RNA abundance in adrenal glands, appendix, brain, and liver; fraction of tissues with detectable RNA expression; and protein abundance in HeLa cells), the positive charge of proteins, the hydrophobicity of proteins, the sensitivity of genes towards mutations (incidence rate of missense mutations in human populations, incidence rate of loss-of-function mutations in human populations, tolerance against homozygous or recessive loss-of-function variation in human populations, CRISPR score in KBM7 cells), the length of the corresponding transcript and gene, and the presence of signal sequences that promote the translocation of nascent proteins into the endoplasmic reticulum. These 15 features are sufficient to account for the model’s accuracy because models using exclusively those features yields prediction accuracies highly comparable to those of the full model when trained on the same 12,948 genes with a complete catalog of features (Spearman: 0.61, S3B Fig), or on all 15,056 genes on which these 15 features are defined (Spearman: 0.59, S3C Fig).

We therefore used these 15 features to define a 15-dimensional space for the 15,056 genes that reflects the correlation between publications and individual features and combinations of distinct features (S3 Table). Clusters of genes within this space were enriched for distinct Gene Ontology annotations and thus known biological roles (Fig 1B, S4 Fig). This initial finding demonstrates that the number of publications on genes can be attributed in a large extent solely to a small set of their physical, chemical, and biological characteristics.

### Past research priorities strongly impact current initiatives

**Fig 2:**
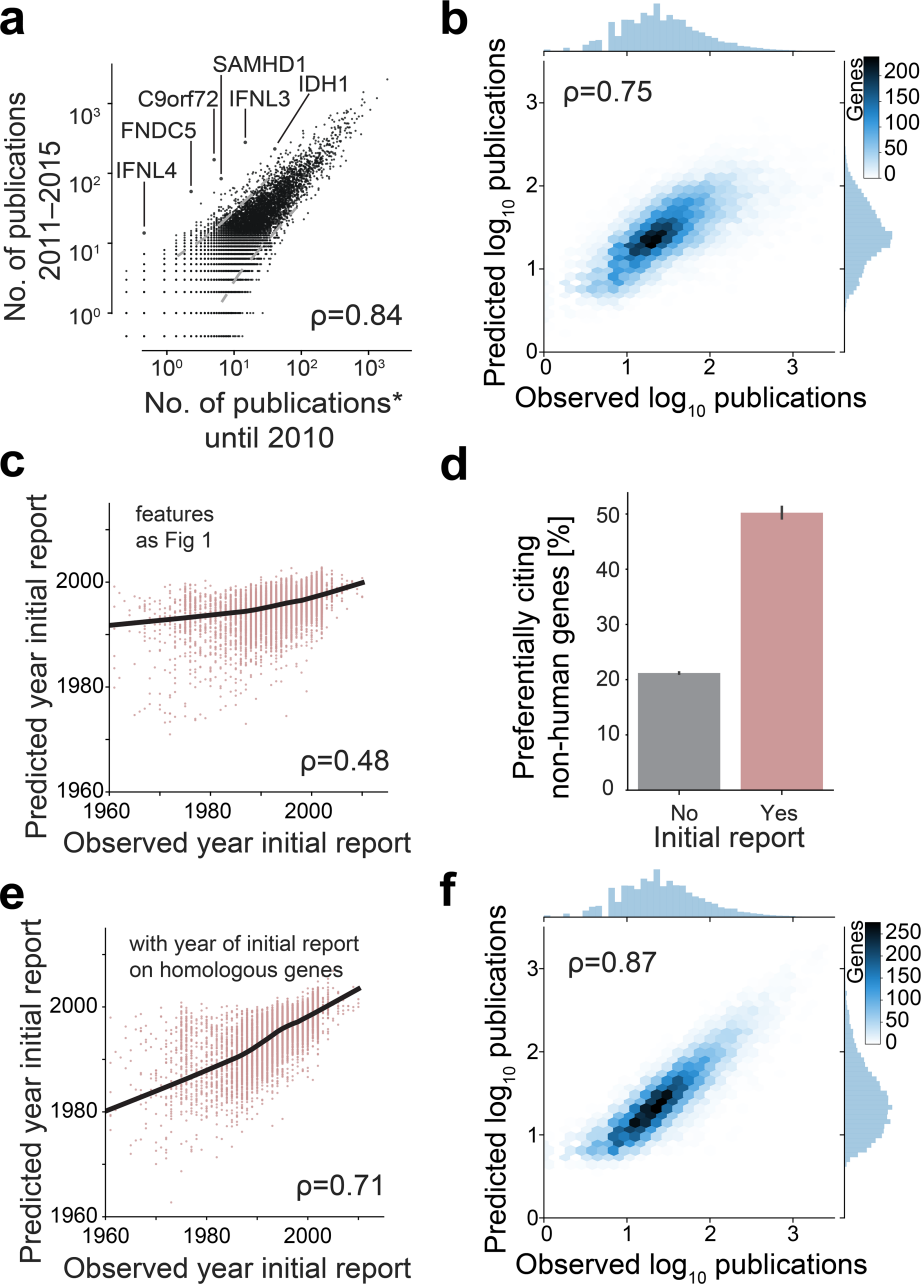
Features of genes and homologous genes predict discovery of human genes. **(A)** Number of publications per gene for past and recent research. Publications of past research (until 2010) are scaled so that the total number of publications matches present research (2011–2015). Dashed grey lines delimit three standard deviations away from the mean. **(B)** Prediction of the number of research publications for the model of Fig 1A extended by including the year of the first publication on the specific human gene (S1 Data). **(C)** Prediction of the year of discovery using the features from Fig 1A (S1 Data). **(D)** Percentage of publications that cite publications with nonhuman genes more frequently than they cite publications with human genes (S1 Data). **(E)** Prediction of the year of initial publications on individual genes using the features from Fig 1A and the year of the initial publication on homologous genes of nonhuman model organisms (S1 Data). **(F)** Prediction of the number of research publications using the features of Fig 1A and the number of publications on homologous genes (S1 Data).

The 15 features described above have all been suspected to affect the ability to study specific genes by traditional methodologies [23, 26–28]. Prompted by this fact and ample sociological observations on science, that the “rich” can get “richer” [9, 29], we next detailed the consensus between the overall number of publications per gene and past research. In line with the similarity among prior reports on the disparity in the number of publications per gene, we found that the present inequality in the number of publications has stayed constant since the year 2000 (S5A and S5B Fig). Similarly, we found the number of publications per gene to be highly correlated between the current decade and preceding time periods of research (Fig 2A, Spearman: 0.84). Interestingly, we also identify six genes that are presently experiencing a strong increase in their number of publications, which can be traced back to a recent acknowledgment of their medical importance (S4 Table).

In contrast to the alternative hypothesis that research patterns on human genes would be particularly dynamic [1, 2], and generalizing beyond earlier studies on two gene families [6, 21] and genes expressed specifically in the brain [30], we find that human genes that had been reported early—as indicated by an early initial publication date on the genes or their encoded gene products [19, 31]—tend to also be more studied presently (S5C Fig, Spearman: 0.58). For example, all genes that had been reported upon by 1991 (corresponding to 16% of all genes) account for 49% of the literature of the year 2015 (S5D Fig). Initial reports further add to the predictability of the number of publications as an inclusion of their year improves the models’ accuracy (Fig 2B, Spearman: 0.75). To identify the factors associated with the initial reports of genes, we next created separate models with the above 430 features and trained them to predict the year of initial publications. While these predictions are slightly less accurate (Fig 2C, Spearman: 0.48) than predictions on the number of publications, the underlying models again selected for highly similar features—most prominently, the presence of signal peptides, the abundance of transcript and protein molecules, and the sensitivity towards mutations (S5E Fig, S5 Table). This shows that characteristics of genes, which have been important for the initial discovery of genes, remain partially correlated with the number of present publications on those genes.

Similarly, we observe that while the number of publications is correlated between the first entry (e.g., AKT1) and the second entry (e.g., AKT2) of a gene family (S5F Fig, Spearman: 0.69), first entries have more publications (Mann–Whitney *U* test: *p*-value < 10^−24^). This demonstrates that even among evolutionary and chemically highly related genes, early initial reports coincide with a higher number of publications (S5F Fig).

### Knowledge from model organisms drives research on human genes

Yet, the reduced prediction accuracy observed for the prediction of the year of the initial report may hint at the presence of another factor or factors that were not included in our curation of 430 gene-intrinsic features. Thus, we performed a bibliometric analysis of PubMed to compare individual publications against the genes contained in the publications that they cite. Focusing on the publications reporting the discovery of new human genes, we found an overrepresentation of publications that cite studies of nonhuman genes (Figs 2D and S6A). Inspecting the organisms of these genes, we observed two classes of organisms. The first class preferentially co-occurred together with human genes and consisted of *Mus musculus*, *Rattus norvegicus*, *Bos taurus*, and *Gallus gallus* (37%, 9.1%, 2.6%, 2.5% of all citations, respectively). The second class preferentially occurred in publications without human genes and consisted of *Drosophila melanogaster*, *Saccharomyces cerevisiae*, *Escherichia coli*, *Xenopus laevis*, *Caenorhabditis elegans*, and *Schizosaccharomyces pombe* (22%, 10%, 4.0%, 2.5%, 1.6%, 1.5% of all citations, respectively) (S6B Fig). Assuming that citations are one proxy of scientific impact, this finding suggests that initial reports on human genes have been particularly influenced by research in model organisms and that multiple model organisms have contributed complementary roles in the discovery of human genes.

With these insights, we dramatically increased the prediction accuracy of the year of initial report of a human gene by including the years of the initial reports on homologous genes of model organisms (Fig 2E, from Spearman: 0.48 to 0.71). Moreover, the years of the initial reports on homologous genes improved prediction accuracy of the number of publications to a greater extent than the year of the initial report on the human genes themselves (S7A Fig, Spearman: 0.81).

Consistent with the picture emerging from these analyses, the homologous genes of unstudied human genes are likewise unstudied in model organisms (S6 Table), and including the number of publications on homologous genes yielded almost perfect predictions of the number of publications for individual human genes (Fig 2F, Spearman: 0.87), while human-specific genes without homologous genes remain significantly less studied (S7B Fig, Mann–Whitney *U* test: *p*-value < 10^−32^). Taken together, these findings demonstrate the impact of research on model organisms on the knowledge acquired on human biology—a hypothesis that had been proposed but not demonstrated previously [32].

### Characteristics of genes affect research on important biology

**Fig 3:**
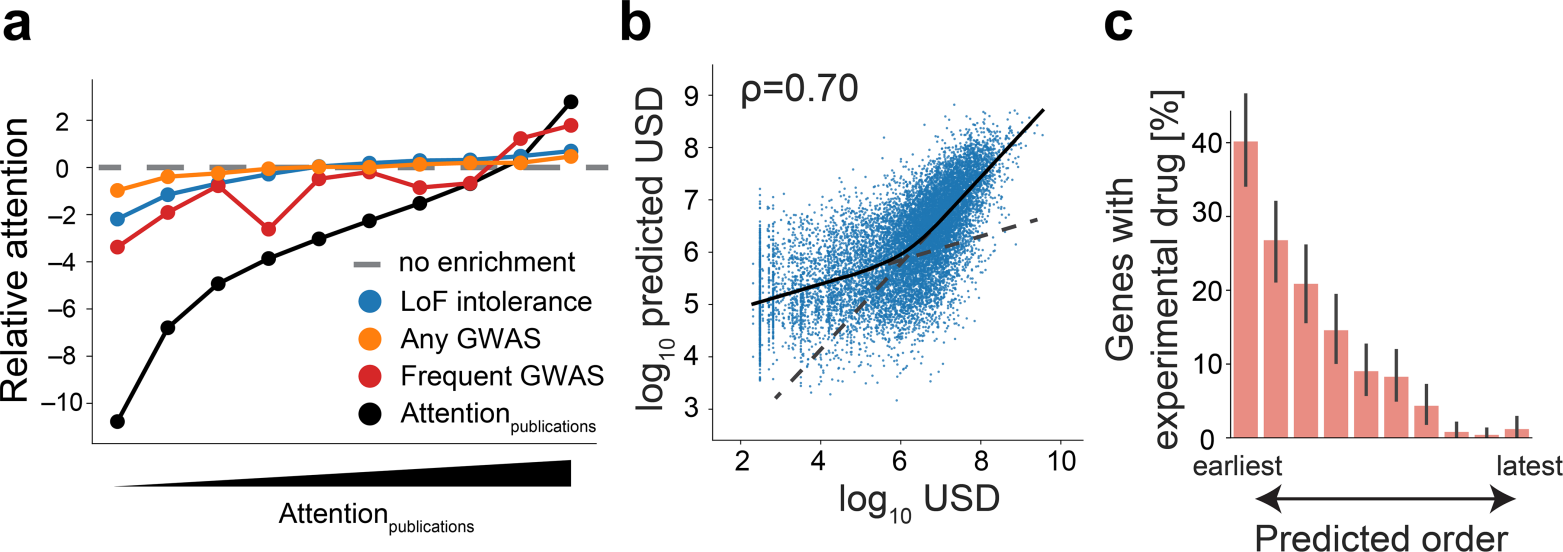
Many potentially important genes are not being studied enough. **(A)** Relative enrichment of the presence of genes with genetic loss-of-function (LoF) intolerance, presence of genes with GWAS traits, and the attention within publications. **(B)** Predicted versus actual NIH budget spending on individual genes (dots). The black line shows a lowess fit and the dashed lines show the two distinct regimes of the prediction (S1 Data). **(C)** Fraction of disease-linked genes with at least one experimental drug conditioned on the predicted order of discovery according to the model shown in Fig 2B. Error bars show 95% confidence intervals for the estimations. GWAS, genome-wide association study; LoF, loss-of-function; NIH, National Institutes of Health; USD, US dollar.

Given the observed historic continuity of scientific endeavors, we wondered whether biomedical research has already identified all particularly important human genes and hence allocates the production of publications accordingly. We follow the naïve assumption that researchers distribute their attention equally across all genes contained in the same publication (S8 Fig). Despite this simplifying assumption, we reassuringly observe that genes that have received the most attention in publications are around three to five times more likely to be sensitive to loss-of-function mutations or to have been identified in genome-wide association studies (GWAS) (Fig 3A). This enrichment is greatest for genes that have been repeatedly identified by several independent studies on the most frequently studied human phenotypic traits. However, we observe an extraordinarily more extreme 13-fold enrichment in the average attention when comparing the genes that have received the least attention to those genes that have received the highest attention (Fig 3A). Hence, while biomedical research does focus on important genes, a disproportionally high amount of research effort concentrates on already well-studied genes.

We observe a similar pattern when inspecting the allocation of funding by the National Institutes of Health (NIH) as another proxy of importance. Although not surprising given the correlation between the number of publications per gene and the amount of funding allocation by the NIH (S9A and S9B Fig, Spearman: 0.95), the above modeling strategy accurately predicts the allocation of billions of research dollars (Fig 3B, Spearman 0.70), and would do so particularly well for genes supported by multiple grants (S9C Fig). Yet, prediction accuracy only marginally improves by additionally considering 3,176 features detailing known annotations between genes and diseases (S9D Fig, Spearman: 0.73), and is greatly—but not completely—impaired if only considering the latter (S9E Fig, Spearman 0.43). This shows that the previously uncovered intrinsic characteristics of genes and the year of the initial report of homologous genes not only correlate with research funding, but that they would do so to a larger extent than presently existent knowledge about the role of genes in disease.

Along the same lines, if exclusively considering genes with a reported role in disease, we found that the same models that had predicted the year of the initial publication of genes (Fig 2E) also predicted the likelihood of the existence of both approved and preclinical drugs (Fig 3C, S9F Fig).

Collectively, these findings show that a small number of characteristics of genes and the availability of model organisms exert a strong influence on basic and applied research on human disease and that the resulting research can significantly deviate from the actual biological importance of individual genes.

### Feasibility of alternative discovery strategies

**Fig 4:**
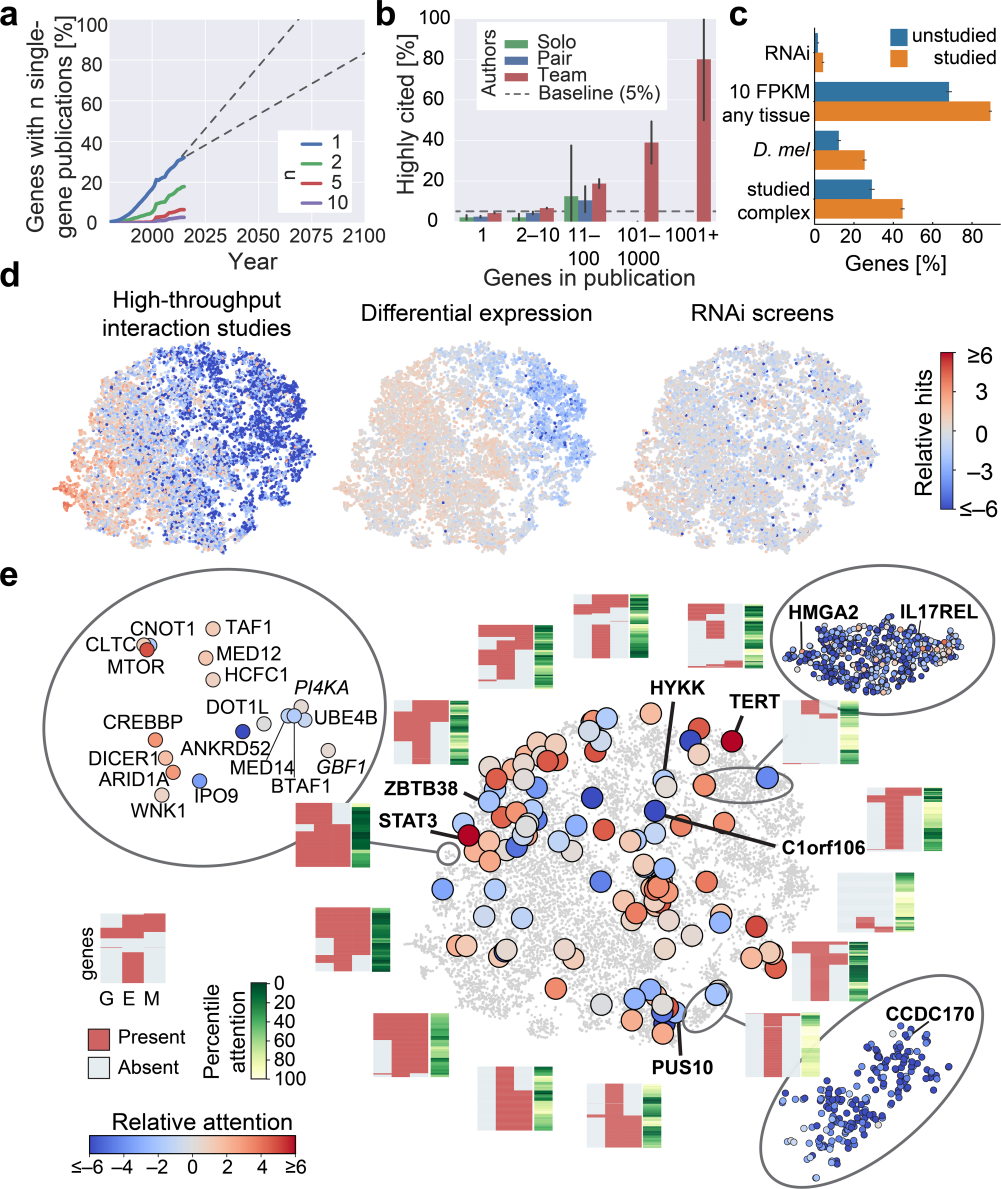
Identifying and exploring ignored genes. **(A)** Estimation of the years until all genes are studied if scientific enterprise continues to follow trends reported above. Number of genes with at least *n* focused (single-gene) publications per year. Dashed lines show extrapolation of the bounds of linear regression for recent years. **(B)** Percentage of highly cited studies (top 5% in number of citations) in the 8 years following their publication. Error bars show 95% confidence intervals. **(C)** Percentage of genes with a strong RNAi phenotype, at least one tissue with moderate RNA abundance, presence of a *Drosophila melanogaster* homolog, or membership in a complex with highly studied genes. Highly studied genes show higher percentages for all these characteristics, but many unstudied genes also share those characteristics. **(D)** Illustration of bias in identification of hits in distinct large-scale experimental approaches. Interaction studies refer to studies labelled as “High throughput” within BioGRID. Relative hits marks fold enrichment over equal occurrence (S1 Data). **(E)** Genes grouped by t-SNE visualization using the 15 features most important to the models used in Fig 1A. Large circles highlight genes with frequently discovered GWAS traits. Heatmaps show presence of strong genetic evidence (G), experimental potential (E), and homolog in invertebrate model organism (M). Note the lack of a strong correlation between GEM characteristics and research attention. E, experimental potential; FPKM, fragments per kilobase of transcript per million mapped reads; G, strong genetic support; GEM, strong genetic support and experimental potential and homolog in invertebrate model organism; GWAS, genome-wide association study; M, model organism; RNAi, RNA interference; t-SNE, t-distributed stochastic neighbor embedding.

The strong correlations uncovered, and earlier work on the availability of reagents [5, 6, 21] suggest, that researchers may face very practical constraints that prevent them from exploring little-studied genes and that there might be a need for alternative discovery strategies [33]. In support of this possibility and extending beyond the above findings on the bulk of accrued knowledge, we observe that the fraction of genes that have been described in focused single-gene studies has only been increasing at a constant rate (Fig 4A). Extrapolating from this trend, we estimate that it would take at least five decades until all genes are sufficiently studied. Similarly, simply studying little-studied genes might not be very informative and could expose junior scientists at an increased career risk (S10A Fig). Along the same lines, grant categories of the NIH dedicated to exploratory research, which do not require preliminary data, and grants categories dedicated to innovative research or the training of scientists all closely reproduce the imbalance observed for the biomedical literature, with 5% of the human protein-coding genes accounting for half of the publications (S10B and S10C Fig). Given a recent bibliometric study, which demonstrated that novelty could, however, be beneficial for the impact of a scientific publication if combined with an established research context [34], we therefore thought to build a resource that provides a context for the exploration of little-studied genes.

Inspecting the properties of existing publications on little-studied genes, we found that these genes tend to occur in large-scale investigations that include most genes (S11A and S11B Fig). Hinting at an ability of large-scale studies to support research on less investigated genes, we observed that these studies serve as a frequent reference for other publications (Fig 4B, S11C Fig) and that single-gene studies that refer to them tend to focus on genes that are less studied than those genes contained in single-gene studies that refer to single-gene studies (S11D Fig).

To determine the extent to which large-scale collections of biological information could already serve as potential starting points for detailed characterizations on most genes, we next extended our resource with databases—such as a collection of public RNA interference (RNAi) experiments [35], a catalog of human protein complexes [36], and a catalog of public differential gene expression experiments [37]—that could potentially be affected by biased experimental choices. We find that the 27% of genes that have never been studied by a full publication (S12A Fig) are less frequently identified in publicly available data of large-scale experiments and that they are less likely to have characteristics associated with a high number of publications (Fig 4C, S12B Fig).

However, we also find that there already exist gene-specific data on possible experimentation for 83% of them and that for 25% of them, there exist at least three qualitatively distinct types of data (S12C Fig). This strongly suggests that the characteristics of genes and homologous genes that prevented their early discovery would no longer prevent their more detailed study.

To facilitate exploration and hypothesis generation, we provide a curated guide that specifically directs to the appropriate sources of gene-specific preliminary data (S7 Table).

Our analysis further shows that distinct large-scale approaches cover distinct areas of the 15-dimensional space, with genes identified in high-throughput interaction studies being strongly enriched in regions containing abundantly expressed genes [23], and genes identified through differential expression studies being enriched in regions containing genes whose transcripts are ubiquitously detected in adult tissues through current technology. In contrast, genes identified through their phenotypes within loss-of-function RNAi screens cover the 15-dimensional space more evenly (Fig 4D). Similarly, genes with a highly reproduced association to genetic traits cover multiple areas of the 15-dimensional space, some predicting a large number of publications and others predicting a small number of publications (Fig 4E, S4 Fig). For illustration, consider the RNA of the heavily studied gene, TERT, the catalytic subunit of telomerase, which is undetectable in most adult tissues. While our analysis shows that this biological characteristic is generally associated with a low number of publications, the absence of TERT restricts excess cell proliferation [38]—a factor that overcomes the difficulty in its study following its ectopic activation. Another interesting illustration is provided by the poorly studied breast cancer gene CCDC170, which encodes for one of the most charged and acidic human proteins but also appears to have some structural role in maintaining the organization of Golgi-associated microtubules [39]. As a final illustration, consider C1orf106, a gene with the second-strongest genetic association to ulcerative colitis. Despite being among the top 20% of genes with the most frequently identified associations in differential gene expression experiments (S7 Table), C1orf106 had never been followed up until recently, when gene-specific pull-down experiments revealed its role in the regulation of the stability of epithelial adherens junctions [40]. This demonstrates that functional studies remain a powerful strategy to discover novel biology that does not reproduce past research biases.

To provide a broader perspective on the strategic options for further exploration, we next introduced aggregate measures for the presence of genetic support and experimental approachability and the existence of homologous genes in invertebrate model organisms. While some of the initially identified clusters (Fig 1B) seem experimentally well accessible in humans or model organisms, other clusters seem resilient to those approaches (Fig 4E). An opposite example is a cluster enriching for transcriptional coactivator activity. It contains several evolutionarily conserved genes that are highly sensitive towards loss-of-function mutations and experimentally approachable. This cluster contains multiple highly studied modulators of cellular physiology, such as the genes MTOR, CLTC, TAF1, and CREBBP. However, this cluster also contains DICER1, which catalyzes the maturation of microRNAs and is a recent recipient of research attention, and whose discovery was perceived as an enormous surprise following a long-held lack of attention towards non–protein-mediated gene regulation [41]. Intriguingly, this cluster includes two still mostly uncharacterized members of large gene families, IPO9 and ANKRD52. This lack of attention illustrates that even genes with seemingly promising characteristics can remain mostly ignored. To facilitate identification of such genes, we are also providing a list of these genes (S8 Table) and a map that identifies them within the vicinity of custom sets of genes (S9 Table). We further add another map that allows probing custom sets of genes for the above aggregate measures (S10 Table).

## Discussion

Because the difficulty of pursuing different research directions varies both within distinct fields of biological and nonbiological inquiry [16], we suspect that our findings may be generalizable to other areas of science. For example, mathematics dealt for centuries nearly exclusively with “smooth” curves; only in the last half century did it address the study of infinitely rough curves [42].

Our work demonstrates that even highly promising genes that could already be studied by current technologies remain ignored. This suggests that the ossification of past research topics [43], which for human genes becomes apparent at the turn of the millennium (S5A and S5B Fig), reflects upon processes that extend beyond past experimental possibilities. Indeed, a recent seminal bibliometric study on 250 scientific fields, including molecular biology, demonstrated that scientific fields move from a phase characterized by “the rich get richer” towards a phase of ossification as the annual number of publications increases [43]. Our study provides empirical support for the presence of several processes that could possibly contribute to this ossification, including but not limited to the availability of prior knowledge [7]; biases in computational annotations; the availability of reagents [6, 21]; the career prospects of junior researchers; the support by grants [3]; training agendas; the presence of an overwhelming set of competing future research options [43]; a slow transition of research between large-scale studies and small-scale studies [44, 45]; a sustained ease to experimentally study certain genes; a shortage of large-scale studies that attribute function through perturbing genes and monitoring altered physiology rather than through guilt by association [46, 47]; and a decrease in the workforce that uses model organisms, which accelerated around the year 2000 in favor of an increased fraction of scientists that exclusively work on human genes (S13 Fig). Similarly, our work shows that, with some rare exceptions, the human genome project did not suffice to promote an exploration of novel genes and the biology encoded by them.

Given their presence in the human genome, it is certain that the majority of protein-coding genes have biological relevance [48]. For some genes the relevance might be apparent, such as for the δ- and β-globins [49, 50], which mark among the first human genomic clones and encode for the hemoglobin subunits. For other genes, most of their physiological relevance might only unfold after their basic characterization outside of medical contexts, such as for the heat shock-inducible gene HSP70, which marks an important subsequent human genomic cloning endeavor [51] and participates in a network of genes that control protein homeostasis—a process whose failure characterizes aging in humans and model organisms, and a basis for diseases of protein conformation [52]. Furthermore, many current insights on biology relate to monogenic experimentation schemes, whereas biological processes appear polygenic, which could plausibly further contribute to the continued inability to explain many of the biological processes known to occur [53]. Indeed, our work supports the hypotheses that an insufficient understanding of the biology of many disease genes has prevented the successful development of therapies [7, 54, 55] and that preclinical research is biased towards experimentally well-accessible genes [28]. To visualize potentially implicit biases underlying distinct research projects and findings, we provide a copy of the 15-dimensional feature space, whose regions correspond to distinct biases (S4 Fig, S3 Table).

In order to accelerate the pace of discovery, we propose the need for funding mechanisms of scientists and calls for proposals that encourage the pursuit of nonredundant and likely highly unpredictable research directions. In order to counter the career forces currently pushing towards conformity, there would be a need for stable, long-term support for such innovators to focus on the unknown. Just as the Royal Society sponsored target studies of the unknown with an eye towards the economic potential of certain discoveries, we also predict that exploring the uncharted territories of unknown biology by investigating unstudied and understudied genes will yield satisfying observations that would contribute economically and medically. We believe that the resource presented here provides a jumping point for further systems-level investigation on the formation of scientific knowledge [56] and a guide to researchers who want to identify promising but little-studied genes.

## Materials and methods

### Data sources

Linkage of genes to publications was obtained from NCBI NIH (https://ftp.ncbi.nlm.nih.gov/gene/DATA/gene2pubmed.gz) in early 2017. Patent data were obtained from Rosenfeld and Mason [57]. Gene Ontologies, mapped to Entrez Gene IDs, were obtained from NCBI in early 2017 (https://ftp.ncbi.nlm.nih.gov/gene/DATA/gene2go.gz). Funding information was obtained from NIH ExPORTER (https://exporter.nih.gov/) in early 2017. Names of genes and chromosomes were obtained from NCBI NIH in early 2017 (https://ftp.ncbi.nlm.nih.gov/gene/DATA/gene_info.gz). Article types and publication titles were obtained from MEDLINE (https://www.nlm.nih.gov/databases/download/pubmed_medline.html) through a local copy of their database in early 2017. Disambiguated authorship information was obtained from Clarivate Analytics.

SwissProt and TrEMBL protein sequences, and mapping tables to Entrez GeneIDs, were obtained from Uniprot in early 2017 (ftp://ftp.uniprot.org/pub/databases/uniprot/current_release/knowledgebase/complete/uniprot_sprot.fasta.gz, ftp://ftp.uniprot.org/pub/databases/uniprot/current_release/knowledgebase/complete/uniprot_trembl.fasta.gz, ftp://ftp.uniprot.org/pub/databases/uniprot/current_release/knowledgebase/idmapping/idmapping_selected.tab.gz). Linkage tables between Entrez Gene IDs and Ensembl Gene IDs were obtained from NCBI NIH in early 2017 (https://ftp.ncbi.nlm.nih.gov/gene/DATA/gene2ensembl.gz). Genes, coding sequences from genomic RNA, and validated RNA sequences were obtained from Genbank (Genome version GRCh38.p10) (ftp://ftp.ncbi.nlm.nih.gov/genomes/all/GCF/000/001/405/GCF_000001405.36_GRCh38.p10) using a manually reviewed definition of reference chromosomes according to https://ncbi.nlm.nih.gov/genome. Allele frequencies in human populations were obtained from the ExAc database [58]. Compartment information and protein abundance were obtained from Itzhak and colleagues [59]. Loss-of-function information in human cell lines was obtained from Blomen and colleagues [60], Hart and colleagues [61], and Wang and colleagues [62]. Thermal stability on proteins was obtained from Leuenberger and colleagues [63]. Transcript abundance in cells and tissues was obtained from the human protein atlas [64]. Transcript stability was obtained from Tani and colleagues [65].

GWAS were obtained from the NHGRI-EBI Catalog v1.0 [37].

A local copy of the Web of Science Database was obtained from Clarivate Analytics (and formerly Thomson Reuters). Homologene Version 68 was obtained from NCBI NIH (https://ftp.ncbi.nlm.nih.gov/pub/HomoloGene). Associations between genes and diseases were obtained from Genecard’s GeneALaCart service (https://genealacart.genecards.org) in early 2017 through successive batch queries with all official human (HUGO) gene symbols. The BioGRID database [66] was obtained from BioGRID (Version BIOGRID-3.4.147).

Drugs and their targets were obtained from DrugBank (Version 5.0.7).

Bioplex 2.0 complexes were obtained from Huttlin and colleagues [36]. GenomeRNAi v17 was obtained from www.genomernai.org. EBI Gene Expression Atlas (GXA) was downloaded in spring 2017 from www.ebi.ac.uk/gxa.

### Data engineering

For genes, we determined the fraction corresponding to every nucleobase, and the combined ratio of cytosine and guanine, and counted the number of all nucleobases. For protein-coding sequences, we additionally determined the fraction corresponding to individual codons and measured the codon bias according to multiple methods [67–70].

For transcripts, we obtained FPKM values from Uhlen and colleagues [64] and additionally determined the fraction of samples with an expression below 1 FPKM analogously as a surrogate for detectable expression [64].

For SwissProt and TrEMBL proteins, we determined the fraction of the primary sequence covered by individual amino acids. Moreover, we used BioPython [71] to determine the fraction of acidic, aromatic, basic, charged, helix affine, hydrophobic, polar, uncharged polar, sheet affine, and turn affine amino acids. We further used BioPython to estimate protein GRAVY, the protein’s isoelectric point, and molecular weight. Additionally, we counted the total amount of amino acids and thus the length of the protein. We used the Python version of RADAR [72] with its default settings to measure the total number of repeats, and the total RADAR score, and the length of the highest scoring repeat. We used SEG [73] (from NCBI’s ftp.ncbi.nlm.nih/pub/) with its default settings to measure the total amount of amino acids, the fraction of the protein residing in low complexity regions, the length of the longest low complexity region, and the fraction of the protein covered by the longest low complexity region, and counted the total number of low complexity regions and the number of low complexity regions longer than 5, 10, 20, and 40 amino acids. We used SignalP [74] with its default settings to determine the presence of a predicted cleavage site, the maximal cleavage score, the presence of at least four transmembrane residues, and the nucleotide position of the mature protein.

### Data imputation

In the absence of measurements on transcript expression and stability, we used −1 to indicate the presence of a low expression.

In the absence of a SwissProt protein entry for a gene, TrEMBL protein entries were used for a given gene. In the absence of measurements on protein localization and stability and protein abundance, we used −1 to indicate the presence of a low expression.

### Mapping of genes and gene products

Information of genes and gene products was mapped to Entrez GeneIDs. Only unambiguous mappings were considered. In the case of multiple entries mapping to a single Entrez GeneID (e.g., multiple transcripts encoded by the same gene), we used the median of the features.

### Reference research publications

Unless specified otherwise (for reviews), we considered publications that were

(a) assigned by MEDLINE to correspond to a “case report,” “classical article,” “clinical trial,” “clinical trial phase I,” “clinical trial phase II,” “clinical trial phase III,” “clinical trial phase IV,” “comparative study,” “historical article,” “journal article,” “meta analysis,” “multicenter study,” “randomized controlled trial,” “twin study,” or “validation study”;

(b) were further not assigned by MEDLINE to also be a “review”; and

(c) were further not occurring in a journal in which 50% or more of all articles were assigned by MEDLINE to be a “review.”

### Reference genes

We considered protein-coding genes of *Homo sapiens* (NCBI taxonomy ID: 9606) that would also contain an official HUGO symbol and be featured in at least one reference research publication.

### Clustering of features used in prediction

Features were z-scored across the genes and clustered using Ward’s method.

### Predictions of the number of publications

We predicted the log10-transformed number of publications and z-scored the features across genes. We used 90% of the genes as training data and predicted the remaining 10%. We performed at least 400 randomizations using randomly chosen subsets without replacement. This corresponds to a number of iterations in which, within initial test runs, we would not observe changes in the pooled readout within the number of digits provided in this publication. We used Scikit-learn’s [25] (version 0.19) Gradient Boosting Regressor with 300 estimators and a Huber loss function. The results of individual randomizations for individual genes were pooled by taking the median.

### Grouping of genes by features

We considered the 15 features with the highest median importance to the gradient boosting regression models. We considered all reference genes for which these 15 features were defined and z-scored every feature separately across these genes. Grouping onto two dimensions was done by Scikit-learn’s implementation of the t-distributed stochastic neighbor embedding [75].

### Gene ontologies

We considered entries to be negating if the qualifier started with NOT, or if the evidence code was “ND.” For temporarily valid, computationally predicted entries, we considered the “IEA” and “RCA” evidence codes. We excluded unmapped entries with the evidence code “–” or “NR.”

### Enrichment analysis of grouped genes

Highlighted groups were chosen manually to reflect areas with higher local concentration. Terms considered for enrichment were non-negating, non-temporary Gene Ontology annotations with mapped evidence. We used an EASE score [76], an observation-corrected variant of Fisher’s exact test, and determined the false discovery rate through Scikit-learn’s implementation of Benjamini and Hochberg’s procedure using an alpha of 0.05 [25].

### Analysis of recent trends

To account for an uneven total number of pairs between genes and publications, when defining the enrichment within recent years, we normalized either time interval to have the same number of pairs between genes and publications.

We performed a manual literature review on genes with the highest log2 fold change in the number of publications, upon filtering for the presence of at least 10 publications in the interval between 2011 and 2015. We performed a manual literature review and citation analysis to identify findings that changed research on those genes in the subsequent years. Genes highlighted in the main figure were chosen manually to cover a broad range of different numbers of publications, while a complete list is given in S4 Table.

### Predictions of the year of the initial publication

The prediction of the year itself was done as described above for the prediction of the number of publications. When adding discoveries of homologous genes, we considered the years of the first description of homologous genes and the years of the first single-gene publications of homologous genes of model organisms listed in Homologene, and indicated absent values (indicative of the absence of either a homologous gene or publications) by assigning the value −1.

### Estimation of confidence intervals in display items

Confidence intervals of 95% reflect bootstrapped estimates as computed by Python’s seaborn package [25] (versions 0.7 and 0.8).

### Citations towards model organisms

We defined publications with a discovery of a new human gene as those publications that would report on a gene within the year in which the first report on the same gene would appear. We counted the number of cited publications that would have at least one human gene, and the number of cited publications that would have at least one nonhuman gene.

### Fractional counting of publications (attention)

For analyses showing the fraction of literature, we performed a fractional counting of publications. Rather than counting every publication as 1 towards every gene, the value of a publication towards a given gene would be 1/(number of genes considered in the publication).

### Analysis of human-specific genes

We considered genes that would not map to a Homologene group with at least one nonhuman gene. The analysis only included genes with a human Entrez Gene ID that would be smaller or equal to the highest human Entrez Gene ID within the Homologene Database and thus could have been considered for Homologene.

### Analysis of attention enrichment

We performed a fractional counting of publications. Enrichment was calculated as the log2 fold change over the (fractional count of publications in indicated time frame) / [(total number of publications in indicated time frame) / (number of reference genes)].

### Analysis of GWAS

We considered EBI’s mapping of associations and only considered associations lying within the sequence of one, but not multiple, genes. We counted the occurrence of at least one association per publication between a gene and a trait. For strong association, we only considered traits covered in at least 10 distinct studies and genes that would be associated with more than 20% of the studies for such a trait.

### Analysis of strong loss-of-function intolerance

We considered genes with a pLI over 0.9—a threshold that the authors [58] describe as “extreme loss-of-function” intolerance on their accompanying web portal.

### Estimation of funding per gene

We considered NIH funding information between 1985, the year in which data of grants would be provided at the resolution of principal investigators, and 2015. We performed inflation correction using the average United States consumer price index. We equally distributed the total money allocated to a given NIH project ID to all publications supported by this project, and subsequently within the individual genes in this project. We used disease associations from Malacards for Unified Diseases, Orphanet, Human phenotypes, and OMIM as disease linkage features and constructed additional features that would count for the total number of entries within each of the four data sets. Because of computational constraints, we subsequently removed disease linkage features with fewer than 10 genes. Notably, prediction accuracy did not improve if keeping all linkages of Unified Diseases (Spearman 0.73 for addition on top of other features—analogously to S9D Fig; Spearman 0.42 for exclusive usage—analogously to S9E Fig) or OMIM (Spearman 0.71 for addition on top of other features—analogously to S9D Fig; Spearman 0.16 for exclusive usage—analogously to S9E Fig).

### Analysis of transitioning to a future principal investigator status

As the rank of the popularity, we used the fractional count of publications up until the indicated year. We only considered publications of authors that have not yet transitioned to a principal investigator status. As principal investigator status, we consider authors that have at least two last author publications with at least one fellow coauthor.

### Mapping of Web of Science to MEDLINE

We matched publications contained in MEDLINE to records from Web of Science in a two-step process:

(a) if available, we used the digital object identifier (DOI), allowing for an unambiguous identification of publication entries;

(b) otherwise, given the MEDLINE record, we retrieve all publications from Web of Science with the same list of authors’ last names, and that were published in the same year and journal. We then identify the best-matching record by calculating the Levenshtein distance (implemented in seatgeek’s FuzzyWuzzy Python package: https://github.com/seatgeek/fuzzywuzzy) between titles of the MEDLINE and the Web of Science record, respectively. We only considered publications that would map unambiguously and had a mapping score of at least 95 (maximum score 100).

In total, for 97% of all publications in MEDLINE containing a reference to a gene, we were able to identify the corresponding record in Web of Science.

### Analysis of fraction of highly cited publications

Following Uzzi and colleagues [34], we counted citations over the 8 years following the year of the publication. Publications with more than two authors and publications with consortium as the sole affiliation were considered to be team publications. For the analysis of BioGRID, we considered BioGRID entries that had been associated with at least one gene in MEDLINE and counted the unique genes of a publication—after pooling the indicated gene A and gene B entries of an interaction—which would usually be indicative of bait and hit, respectively.

### Analysis of experimentation

Western blots following affinity purification were obtained from BioGRID. For differential gene expression analysis, we used EBI GXA and considered genes to be differentially expressed if their (nonadjusted) *p*-value would be below 0.0001. For RNAi, we only considered phenotypes that were not measured through distinct shRNA abundance and only considered genes occurring in at least 20 studies (which could possibly have monitored distinct phenotypes). We considered a gene to have a strong RNAi if more than 30% of the studies containing the gene would report a phenotype for this gene. This was motivated by the (not shown) observation that genes fall into a bimodal distribution according to the fraction of studies reporting a phenotype, separated at the chosen threshold of 30%.

### Code availability

Code for the curation of data sets and for analysis is available at github.com/tstoeger/plos_biology_2018_ignored_genes.

## Supporting information

S1 Fig Extreme inequality in the research attention given to human protein-coding genes. **(A)** Frequency of the number of research publications associated with human protein-coding genes in MEDLINE. Black line shows a log-normal fit to the data (S1 Data). **(B)** Human-curated GO annotations for individual genes, binned by number of publications. Upper limit of nonoverlapping bins is indicated. Error bars show 95% confidence intervals over bootstraps (S1 Data). **(C)** As **B**, but for temporary computationally predicted GO annotations, which are not yet reviewed by a human curator as of spring of 2017 (S1 Data). **(D)** As **B**, but for gene names (S1 Data). **(E)** As **B**, but for gene symbols. **(F)** Presence of patent claims: fraction of genes with at least one patent, binned as in **B** (S1 Data). GO, Gene Ontology.
(TIF)

S2 Fig Catalog of absence of features. **(A)** Hamming-clustering of genes according to absence of features (S1 Data). **(B)** Number of research publications for genes with and without complete catalog of features.
(TIF)

S3 Fig Physical, chemical, and biological features of genes predict the number of publications. **(A)** Ward-clustering of feature importance of 500 gradient boosting regression models. Numbers in brackets indicate order of features in heatmaps in Fig 1B. **(B)** Prediction of the number of publications for the 12,948 genes with a complete catalog of features using the 15 features highlighted in A (S1 Data). **(C)** As B, but for all 15,056 genes for which the 15 features had been reported. FPKM, fragments per kilobase of transcript per million mapped reads; GRAVY, grand average of hydropathy.
(TIF)

S4 Fig Physical, chemical, and biological features mapped to individual genes. z-score of individual features for genes in the tSNE mapping of Fig 1. Numbers in brackets indicate order of features in heatmaps in Fig 1 (S1 Data). tSNE, t-distributed stochastic neighbor embedding.
(TIF)

S5 Fig Predictability of research effort. **(A)** Cumulative share of publications in MEDLINE covered by the fraction of most common genes in decreasing order (S1 Data). **(B)** Gini coefficient (a measure of inequality) for genes in publications over time. When looking at income or wealth, Gini coefficients of 0.6 are considered extreme (S1 Data). **(C)** Correlation between the year of the initial publication on a gene and the amount of publications between 2006 and 2015 (S1 Data). **(D)** Cumulative share of research published in MEDLINE in the year 2015 on genes ranked according to year of initial publications (S1 Data). **(E)** Comparison of median feature importance for predictions of the number of publications and predictions of the year of the discovery (S1 Data). **(F)** Comparison of the number of publications for the first and second member of a gene family for genes for which the name of the family is part of the official gene name (e.g., AKT1 and AKT2) (S1 Data).
(TIF)

S6 Fig Publications reporting the discovery of new genes preferentially cite model organism. **(A)** As Fig 2D, but for individual years during the 1980s and 1990s, the decades in which most human genes were discovered. Also see S5D Fig (S1 Data). **(B)** Fraction of nonhuman organisms cited by initial publications of human genes. Enrichment represents log2 ratio of the fraction of nonhuman organisms among all initial publications on human genes over the fraction of nonhuman organisms among initial publications on human genes, which also cite publications on human genes. The 10 most cited organisms are shown (S1 Data).
(TIF)

S7 Fig Study of homologous genes predicts study of human genes. **(A)** Prediction of the number of research publications using the model of Fig 1A, extended to include the year of the initial publications on homologous nonhuman genes (S1 Data). **(B)** Number of publications for individual genes conditioned on the existence of homologous genes in nonhuman model organisms (human-exclusive). *p*-value: Mann–Whitney *U* test (S1 Data).
(TIF)

S8 Fig Attention in publications closely tracks number of publications. Fractional counting, in which the occurrence of a gene in a publication counts as 1/(number of genes in publication), versus normal counting, in which the occurrence of a gene in a publication counts as 1, of publications with multiple genes (S1 Data).
(TIF)

S9 Fig Health research funding correlates with the number of publications. **(A)** The number of grants for genes as a function of the number of publications on a gene. **(B)** Correlation between the attention of NIH-sponsored research publications and the amount of allocated NIH budget on individual genes (dots). The latter is approximated by equal allocation of project resources to publications and subsequently the genes contained within them (S1 Data). **(C)** The number of grants for genes with indicated levels of total funding. X-axis shows upper limits of nonoverlapping bins. **(D)** Prediction of NIH budget spending on individual genes (dots) upon adding associations between genes and diseases to features considered in Fig 3B. Black line shows lowess fit and dashed lines two distinct regimes of the prediction (S1 Data). **(E)** Prediction of NIH budget spending on individual genes (dots) when considering only associations between genes and diseases. Black line shows lowess fit and dashed lines two distinct regimes of the prediction (S1 Data). **(F)** As Fig 3C, but for approved drugs. NIH, National Institutes of Health.
(TIF)

S10 Fig Career rewards disfavor novelty. **(A)** Career prospects of junior scientists correlate with the preceding attention directed towards genes: probability to transition to principal investigator (PI) status for authors of publications, according to the median attention of the genes in these publications. If, in the preceding years, this attention fell into the quintile of all genes that had received the least attention, the authors have a lower empirically observed chance to have become a PI. This reduction is largely diminished when comparing authors of publications for which the median attention fell into the central quintile of all genes (corresponding to the genes with the 40%–60% most attention) to those authors of publications for which the median attention fell into the quintile of the genes with the most attention (S1 Data). **(B)** Share of MEDLINE published within indicated year that covers the 5% most-studied genes until the indicated year. For R01, Impact and innovation, Exploratory, and Training grant categories, the share of MEDLINE with support of at least one grant of the respective category is compared against the 5% of genes most studied, irrespective of their grant support. **(C)** Illustration of the 1,000 genes occurring in the most publications supported by exploratory grants of the NIH in the year 2015. NIH, National Institutes of Health; PI, principal investigator; R01, Research Project Grant.
(TIF)

S11 Fig Large-scale studies are a reference for many other publications. **(A)** Kernel-density estimation of the fraction of genes with a given number of publications versus the median number of genes co-occurring in the respective publications. The observed pattern is consistent with the notions of “small science” and “big science” (S1 Data). **(B)** Median percentile of attention for publications as a function of the number of genes associated with the publication (same bins as in Fig 4B). **(C)** Percentage of highly cited publications (top 5%, shown with dashed line) as a function of the number of genes associated with a publication in BioGRID (rather than by MEDLINE). Error bars show 95% confidence interval. **(D)** Median percentile of the attention given a single-gene publication as a function of the number of genes associated with the publications it cites.
(TIF)

S12 Fig What we know about poorly studied genes. **(A)** Distribution of the attention (measured by fractional publications) in publications given to genes. Genes with attention levels below 1 are denoted unstudied (blue), whereas genes with attention levels above 1 are denoted studied (orange). **(B)** Percentage of genes with indicated characteristic. **(C)** As **B**, but grouped for the presence of at least one of the characteristics of **B**. Same order as **B**.
(TIF)

S13 Fig Decrease in the fraction of scientists working on model organisms. Fraction of scientists who—within the indicated year—publish exclusively on nonhuman genes (or gene products) or exclusively on human genes (or gene products), or both. The fraction of scientists who exclusively published on human genes had been stable in the 1980s and 1990s, while the fraction of scientists working on human and nonhuman genes has been steadily decreasing at the expense of scientists publishing exclusively on nonhuman genes. Around the year 2000, the fraction of scientists working on human and nonhuman genes started to plateau, while the fraction of scientist working exclusively on human genes increased by approximately 10 percent points and has since been steadily increasing (S1 Data).
(TIF)

S1 Data Sharable data. Data used for the creation of figures and supplemental figures that can be shared without violating restrictions of external public and commercial data sources. For a complete record of all data sets used in the present meta-study, see Materials and methods.
(XLSX)

S2 Data Mapping of PubMed IDs to Web of Science IDs. Mapping of PubMed IDs to Web of Science IDs for publications linked to genes.
(XLSX)

S1 Table List of genes with an incomplete catalog of features. NCBI gene identifiers (Entrez genes), NCBI gene symbols, and Ensemble Gene IDs are provided. NCBI, National Center for Biotechnology Information.
(XLSX)

S2 Table List of features. z-scored values of 433 features (columns) over all 12,948 genes (rows), with a complete catalog of features. (XLSX)

S3 Table Map of the 15-dimensional space. Coordinates of genes in Fig 1B. In addition, the inferred number of publications, NCBI gene symbols, and Ensemble Gene IDs are provided. NCBI, National Center for Biotechnology Information.
(XLSX)

S4 Table Literature survey of genes with increased attention between 2011 and 2015. Enrichment in publications per gene between 2011 and 2015 over the time until 2010. The count of publications until 2010 has been normalized such that the total number of publications matches the time between 2011 and 2015.
(XLSX)

S5 Table Comparison of feature importance for prediction of the year of initial publication and the total number of publications. Median importance of features over 500 independent randomizations of the models for predicting the number of publications and the year of their discovery.
(XLSX)

S6 Table Fraction of unstudied homologs. Number and fraction of unstudied homologs of unstudied human genes for different taxa. Unstudied genes were defined as in S12 Fig and marking genes that have not been covered by the research effort corresponding to a single single-gene study.
(XLSX)

S7 Table Gene-specific context for further exploration of genes. Gene-specific information to facilitate further experimentation. Tissue and cell line with highest RNA expression (“highest tissue,” “highest cells”); flag indicating whether frequently differentially expressed in EBI-GXA (https://www.ebi.ac.uk/gxa); flag indicating whether frequently reported as a hit in RNAi experiments (http://www.genomernai.org); flag indicating whether used for affinity western blots, indicative of functional antibodies (https://thebiogrid.org); invertebrate and vertebrate model with highest number of publications; phenotype frequently reported in GWAS annotation as in Figs 3A and 4E; least- and most-studied genes in same Bioplex 2.0 complex (http://bioplex.hms.harvard.edu); biophysical features for which the gene falls into the top percentile; presence of a protein domain of unknown function; and protein localization as reported by Itzhak and colleagues, 2016, eLife (CC BY). EBI, European Bioinformatics Institute; GWAS; genome-wide association study; GXA, Gene Expression Atlas; RNAi, RNA interference.
(XLSX)

S8 Table Accessible important genes that are studied less than expected. Genes with characteristics that have occurred in fewer publications than predicted by models of Fig 1A and carry the three favorable strategic properties described in Fig 4E (strong loss-of-function sensitivity and GWAS associations, experimental approachability, and the presence of invertebrate model organisms for genes in 15-dimensional feature space). GWAS, genome-wide association study.
(XLSX)

S9 Table Nearby accessible important genes that are studied less than expected. Closest gene of S8 Table for every other gene in the 15-dimensional feature space in Fig 1B.
(XLSX)

S10 Table Accessible important genes. List of genes that have strong loss-of-function sensitivity and GWAS associations, experimental approachability, and the presence of invertebrate model organisms for genes in 15-dimensional feature space. GWAS, genome-wide association study.
(XLSX)

## Abbreviations

CRISPR: Clustered Regularly Interspaced Short Palindromic Repeats
DOI: digital object identifier
EBI: European Bioinformatics Institute
FPKM: fragments per kilobase of transcript per million mapped reads
GWAS: genome-wide association study
GXA: Gene Expression Atlas
NIH: National Institutes of Health
OMIM: Online Mendelian Inheritance in Man
RNAi: RNA interference
SRP: signal recognition particle
t-SNE: t-distributed stochastic neighbor embedding
