# Supplementary figures and images for: Large-scale investigation of the reasons why potentially important genes are ignored
Source: PLoS Biol. 2018 Sep 18;16(9):e2006643. doi: 10.1371/journal.pbio.2006643 (PMC6143198; doi:10.1371/journal.pbio.2006643)

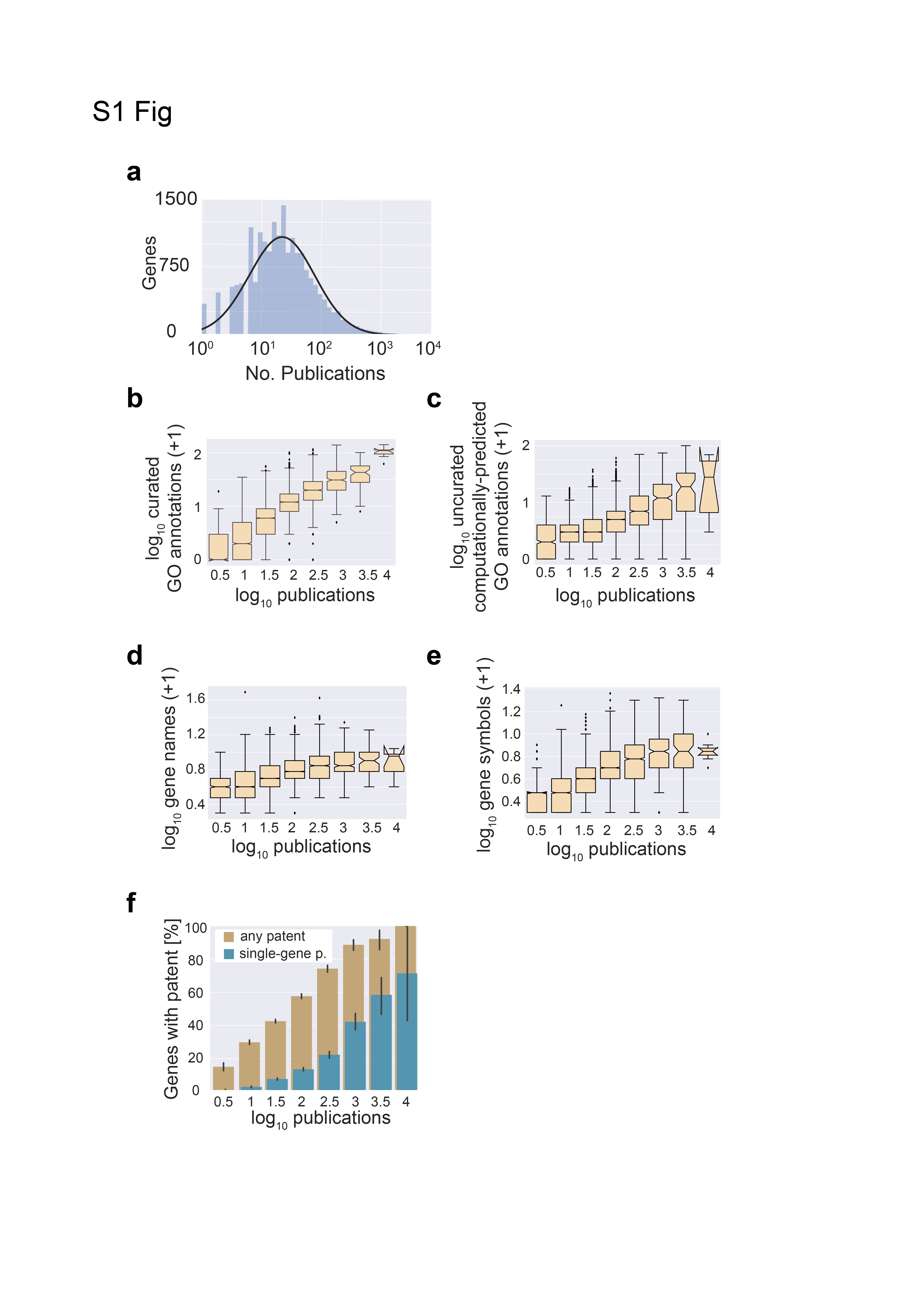

Supplement: S1 Fig — (A) Frequency of the number of research publications associated with human protein-coding genes in MEDLINE. Black line shows a log-normal fit to the data (S1 Data). (B) Human-curated GO annotations for individual genes, binned by number of publications. Upper limit of nonoverlapping bins is indicated. Error bars show 95% confidence intervals over bootstraps (S1 Data). (C) As B, but for temporary computationally predicted GO annotations, which are not yet reviewed by a human curator as of spring of 2017 (S1 Data). (D) As B, but for gene names (S1 Data). (E) As B, but for gene symbols. (F) Presence of patent claims: fraction of genes with at least one patent, binned as in B (S1 Data). GO, Gene Ontology. (TIF) [file pbio.2006643.s001.tif]

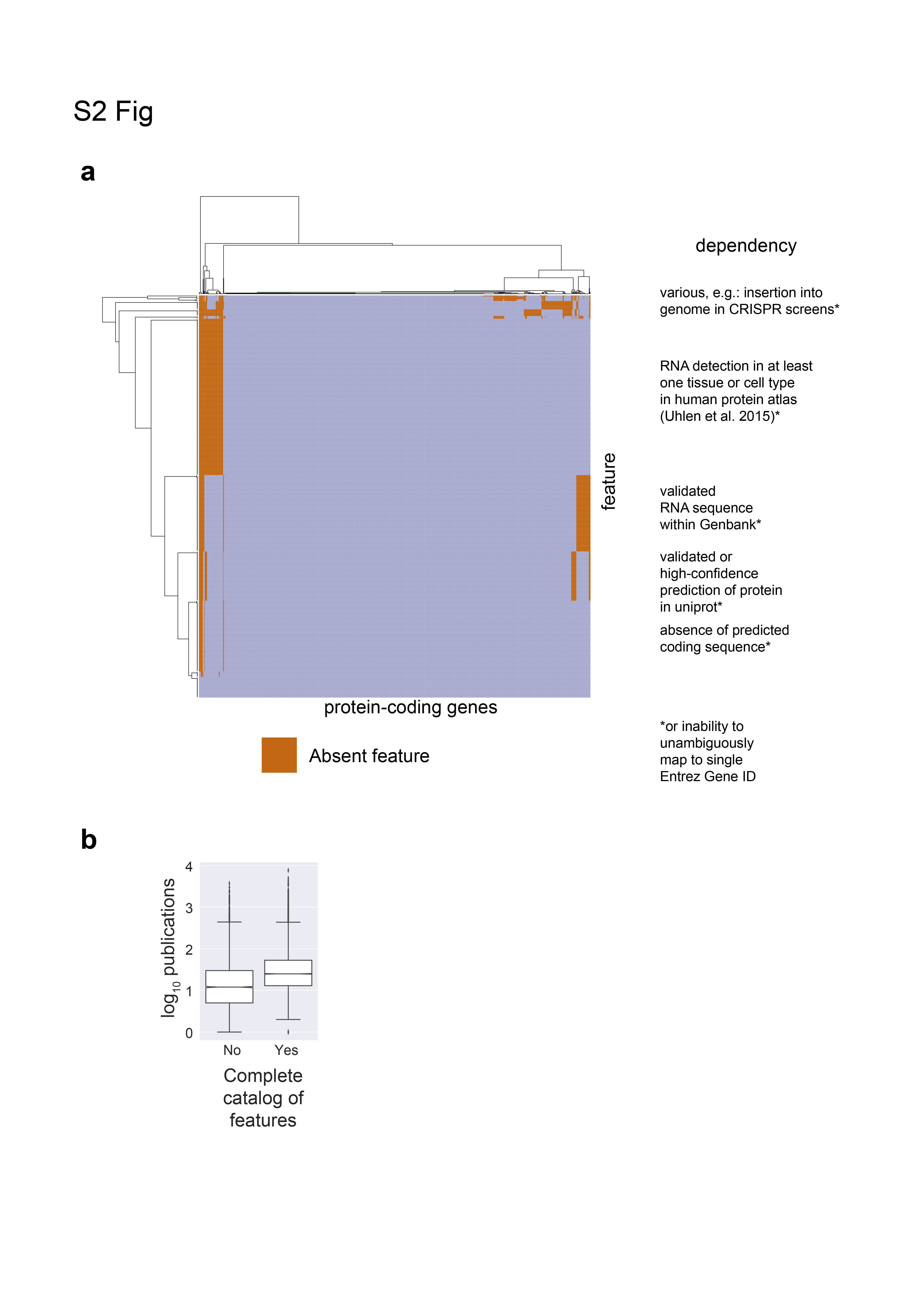

Supplement: S2 Fig — (A) Hamming-clustering of genes according to absence of features (S1 Data). (B) Number of research publications for genes with and without complete catalog of features. (TIF) [file pbio.2006643.s002.tif]

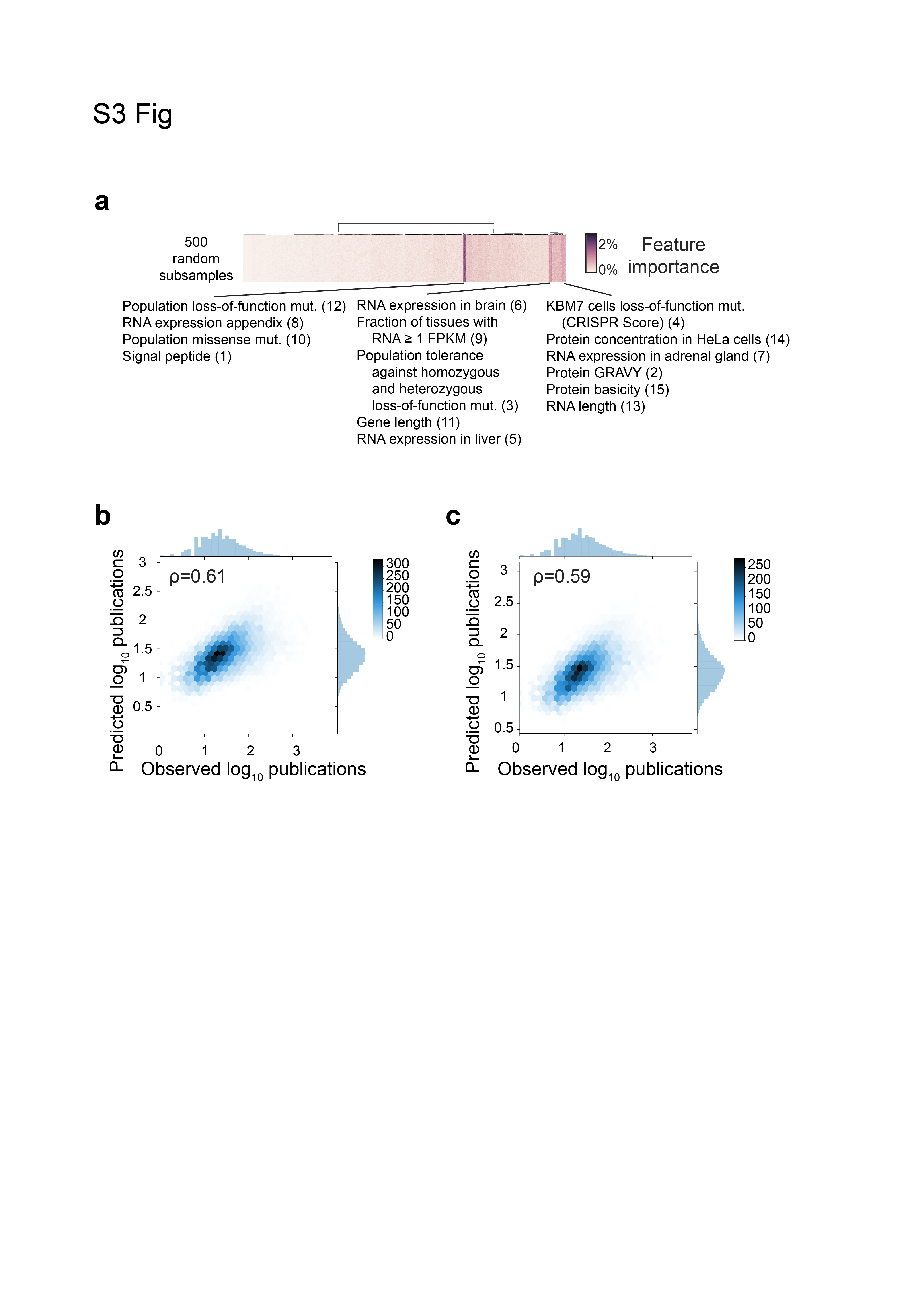

Supplement: S3 Fig — (A) Ward-clustering of feature importance of 500 gradient boosting regression models. Numbers in brackets indicate order of features in heatmaps in Fig 1B. (B) Prediction of the number of publications for the 12,948 genes with a complete catalog of features using the 15 features highlighted in A (S1 Data). (C) As B, but for all 15,056 genes for which the 15 features had been reported. FPKM, fragments per kilobase of transcript per million mapped reads; GRAVY, grand average of hydropathy. (TIF) [file pbio.2006643.s003.tif]

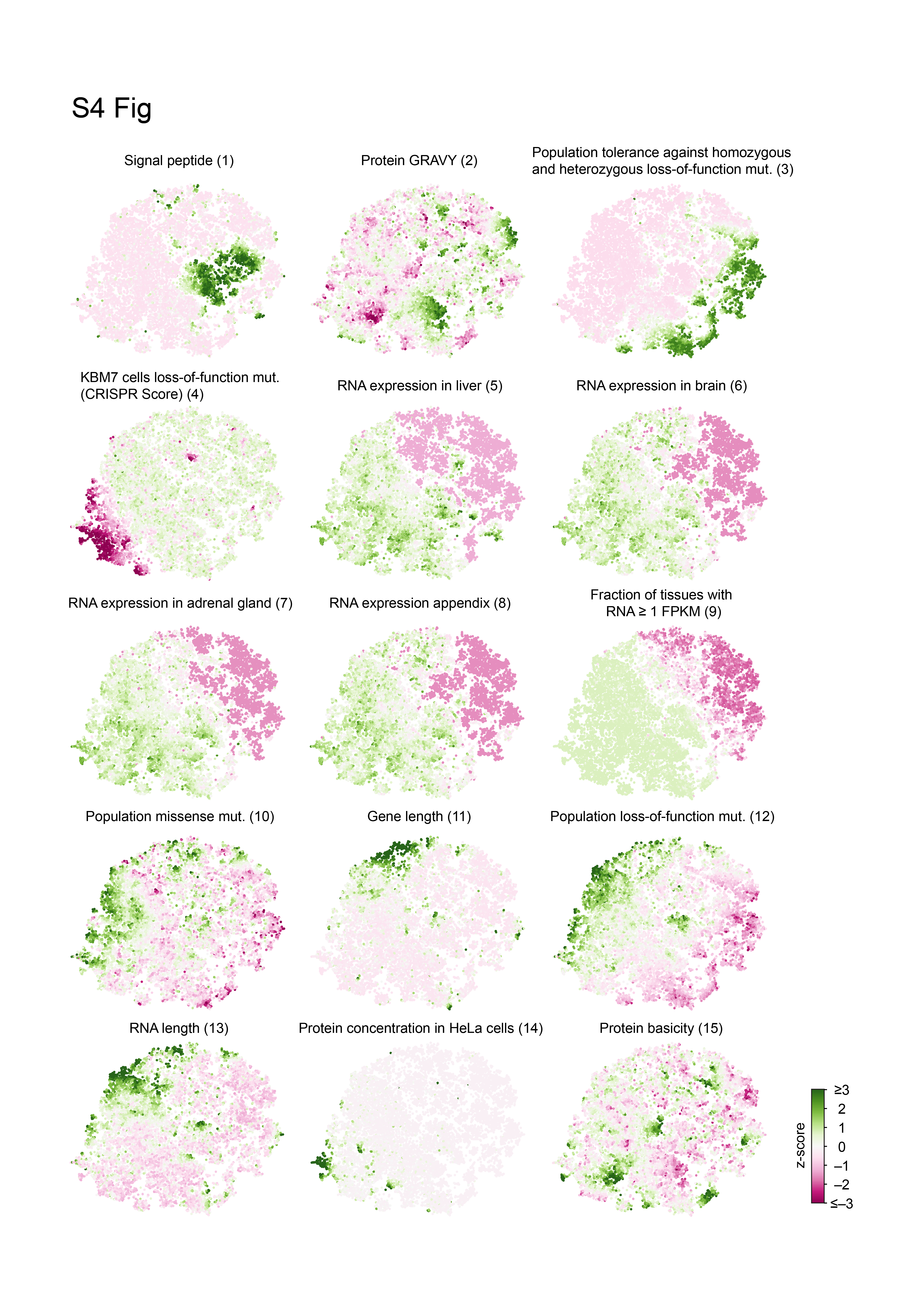

Supplement: S4 Fig — z-score of individual features for genes in the tSNE mapping of Fig 1. Numbers in brackets indicate order of features in heatmaps in Fig 1 (S1 Data). tSNE, t-distributed stochastic neighbor embedding. (TIF) [file pbio.2006643.s004.tif]

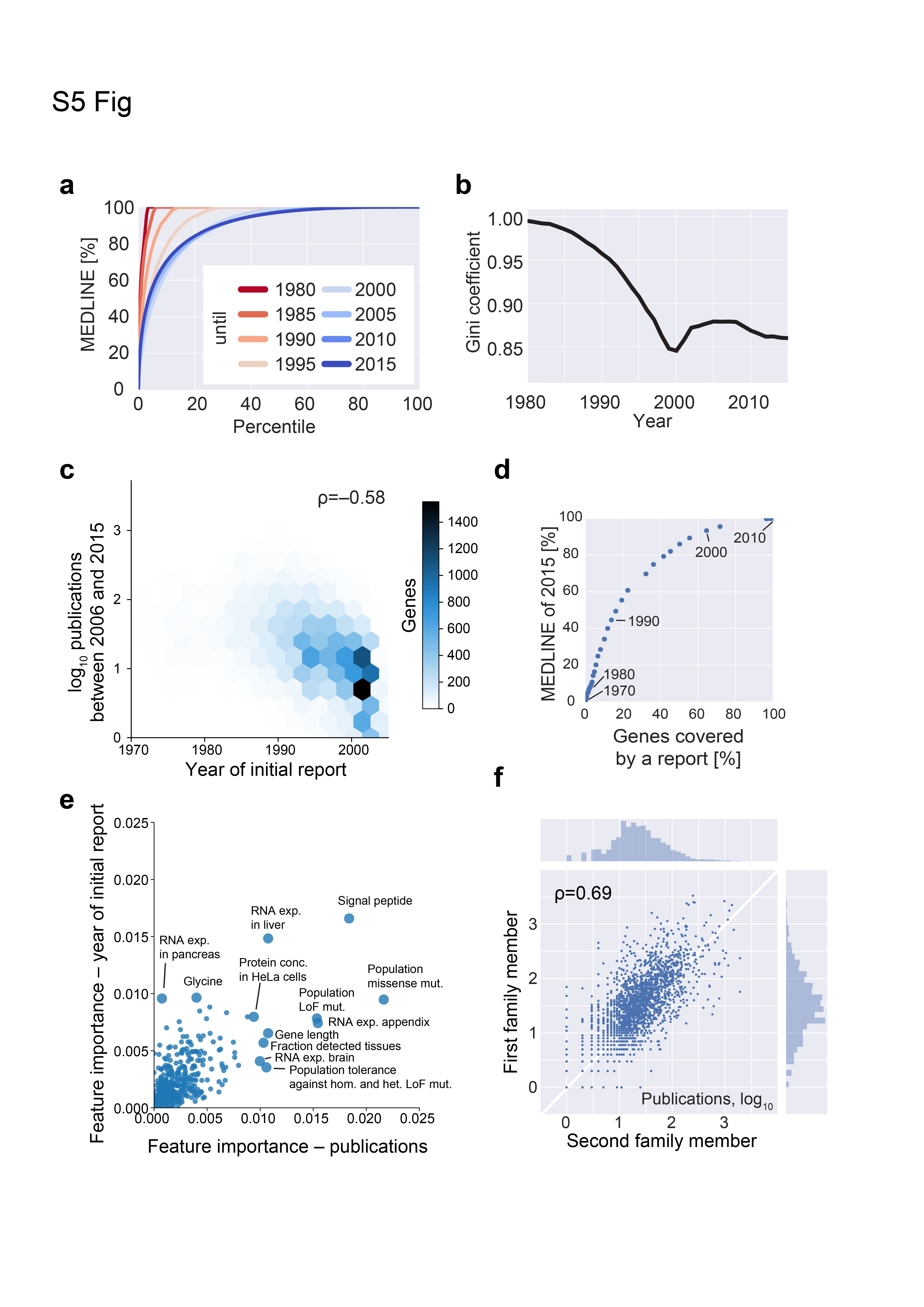

Supplement: S5 Fig — (A) Cumulative share of publications in MEDLINE covered by the fraction of most common genes in decreasing order (S1 Data). (B) Gini coefficient (a measure of inequality) for genes in publications over time. When looking at income or wealth, Gini coefficients of 0.6 are considered extreme (S1 Data). (C) Correlation between the year of the initial publication on a gene and the amount of publications between 2006 and 2015 (S1 Data). (D) Cumulative share of research published in MEDLINE in the year 2015 on genes ranked according to year of initial publications (S1 Data). (E) Comparison of median feature importance for predictions of the number of publications and predictions of the year of the discovery (S1 Data). (F) Comparison of the number of publications for the first and second member of a gene family for genes for which the name of the family is part of the official gene name (e.g., AKT1 and AKT2) (S1 Data). (TIF) [file pbio.2006643.s005.tif]

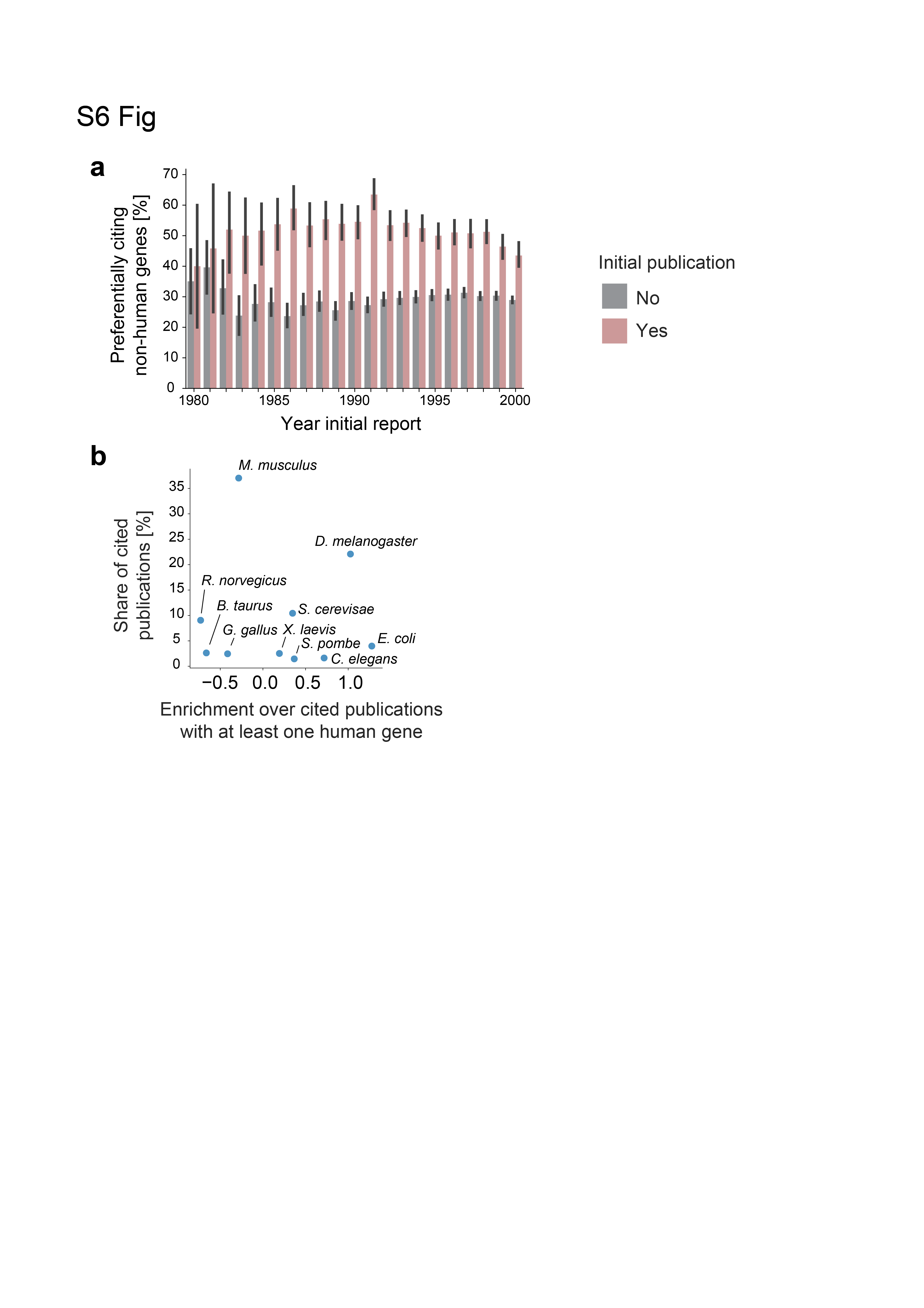

Supplement: S6 Fig — (A) As Fig 2D, but for individual years during the 1980s and 1990s, the decades in which most human genes were discovered. Also see S5D Fig (S1 Data). (B) Fraction of nonhuman organisms cited by initial publications of human genes. Enrichment represents log2 ratio of the fraction of nonhuman organisms among all initial publications on human genes over the fraction of nonhuman organisms among initial publications on human genes, which also cite publications on human genes. The 10 most cited organisms are shown (S1 Data). (TIF) [file pbio.2006643.s006.tif]

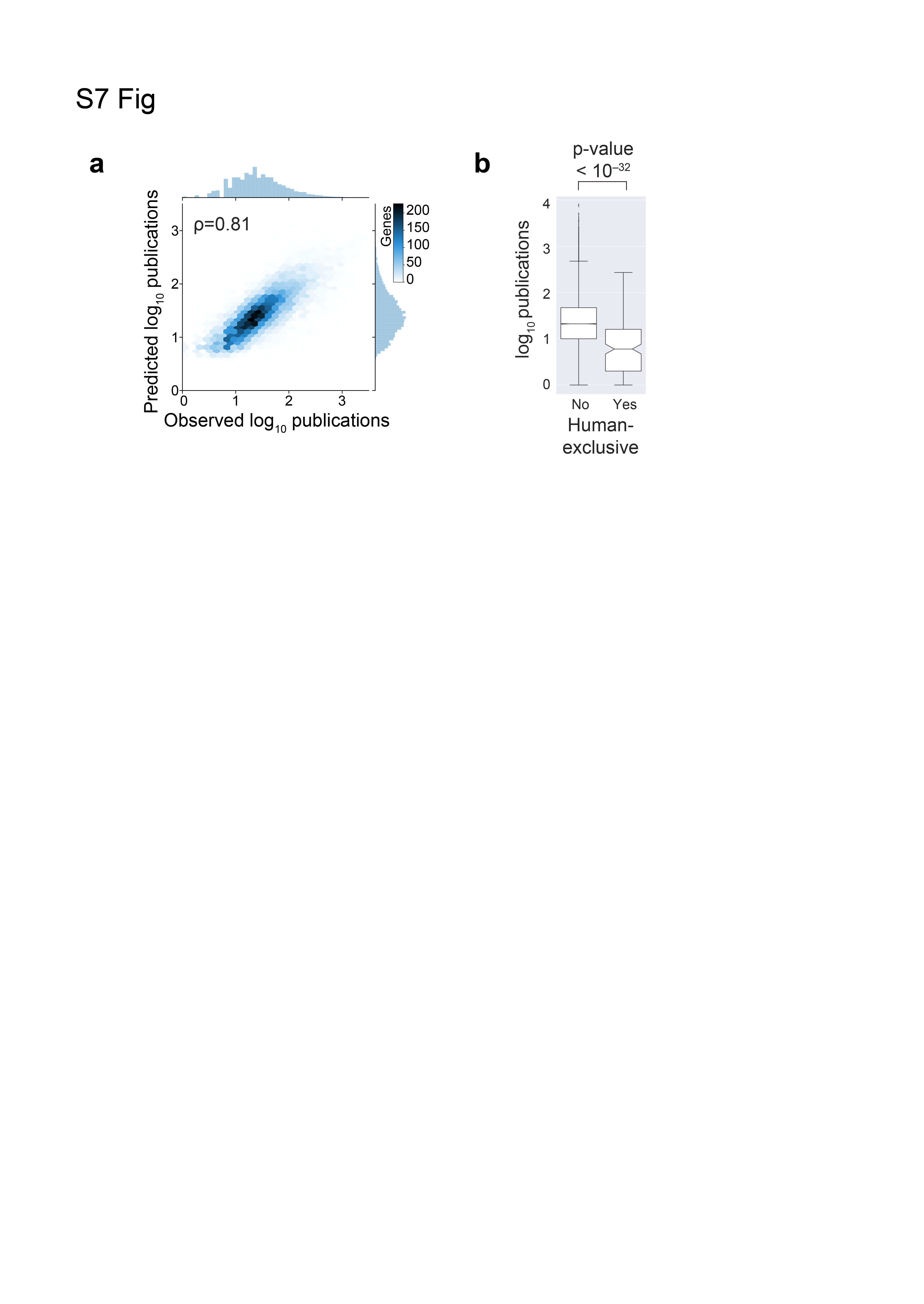

Supplement: S7 Fig — (A) Prediction of the number of research publications using the model of Fig 1A, extended to include the year of the initial publications on homologous nonhuman genes (S1 Data). (B) Number of publications for individual genes conditioned on the existence of homologous genes in nonhuman model organisms (human-exclusive). p-value: Mann–Whitney U test (S1 Data). (TIF) [file pbio.2006643.s007.tif]

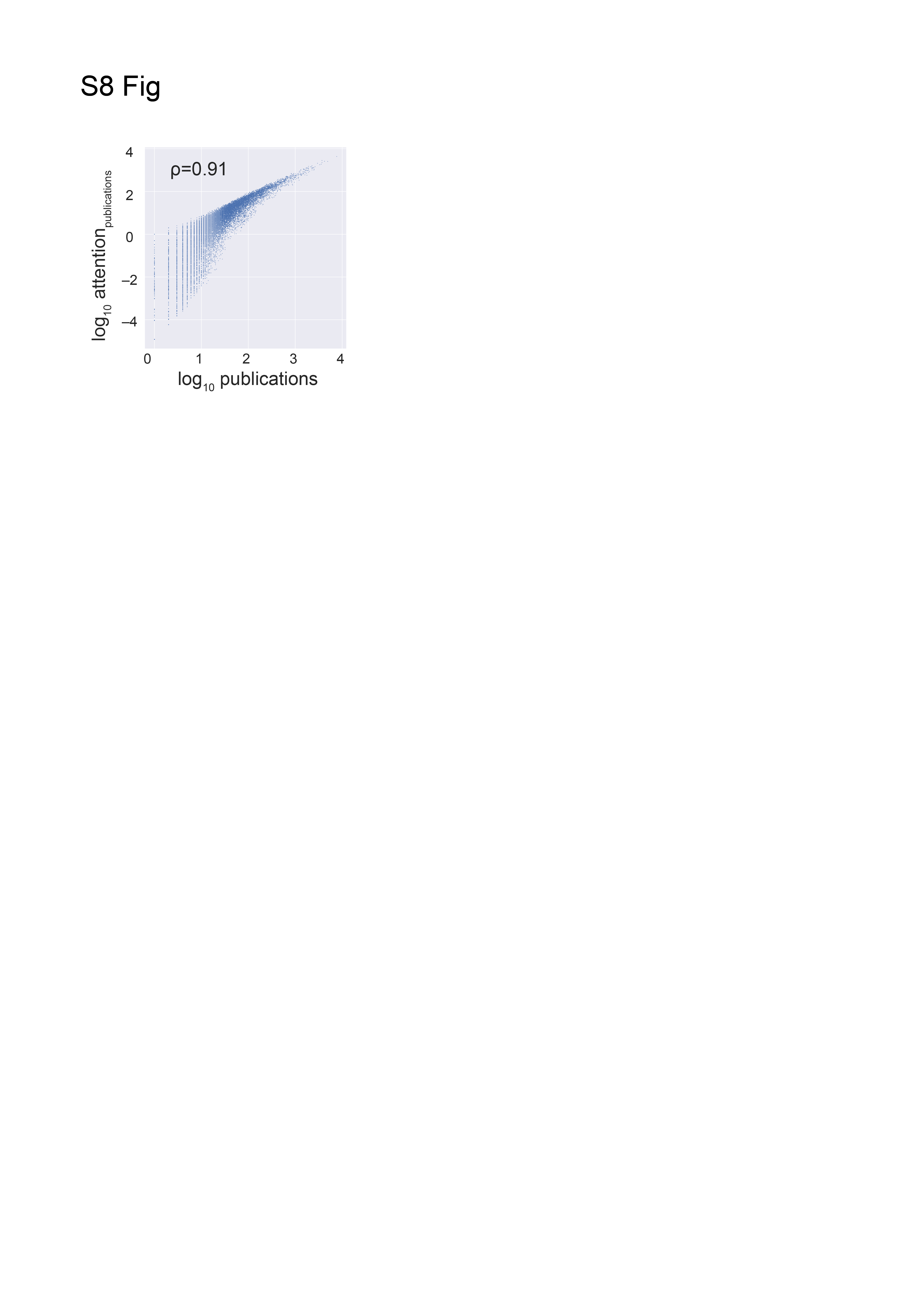

Supplement: S8 Fig — Fractional counting, in which the occurrence of a gene in a publication counts as 1/(number of genes in publication), versus normal counting, in which the occurrence of a gene in a publication counts as 1, of publications with multiple genes (S1 Data). (TIF) [file pbio.2006643.s008.tif]

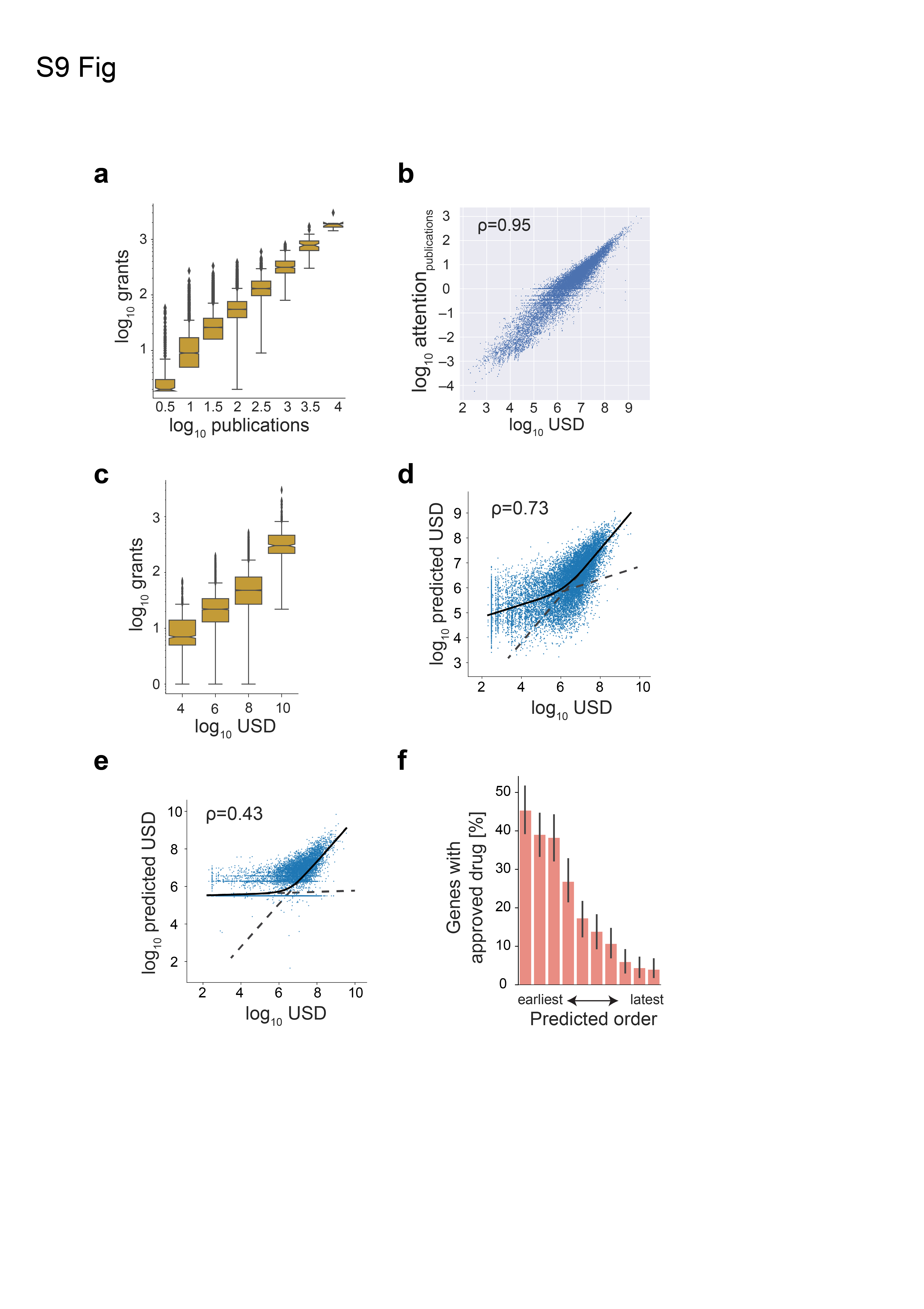

Supplement: S9 Fig — (A) The number of grants for genes as a function of the number of publications on a gene. (B) Correlation between the attention of NIH-sponsored research publications and the amount of allocated NIH budget on individual genes (dots). The latter is approximated by equal allocation of project resources to publications and subsequently the genes contained within them (S1 Data). (C) The number of grants for genes with indicated levels of total funding. X-axis shows upper limits of nonoverlapping bins. (D) Prediction of NIH budget spending on individual genes (dots) upon adding associations between genes and diseases to features considered in Fig 3B. Black line shows lowess fit and dashed lines two distinct regimes of the prediction (S1 Data). (E) Prediction of NIH budget spending on individual genes (dots) when considering only associations between genes and diseases. Black line shows lowess fit and dashed lines two distinct regimes of the prediction (S1 Data). (F) As Fig 3C, but for approved drugs. NIH, National Institutes of Health. (TIF) [file pbio.2006643.s009.tif]

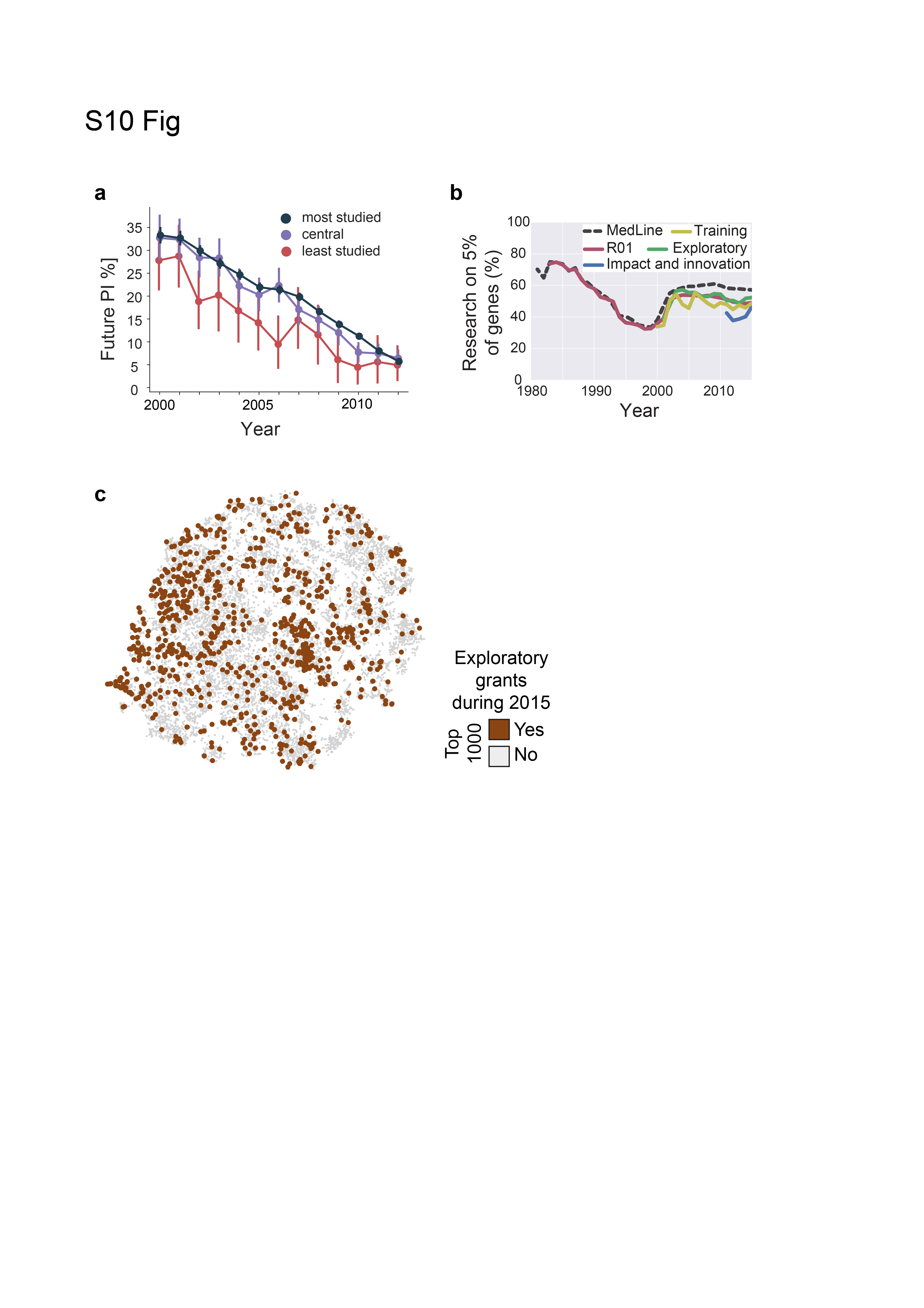

Supplement: S10 Fig — (A) Career prospects of junior scientists correlate with the preceding attention directed towards genes: probability to transition to principal investigator (PI) status for authors of publications, according to the median attention of the genes in these publications. If, in the preceding years, this attention fell into the quintile of all genes that had received the least attention, the authors have a lower empirically observed chance to have become a PI. This reduction is largely diminished when comparing authors of publications for which the median attention fell into the central quintile of all genes (corresponding to the genes with the 40%–60% most attention) to those authors of publications for which the median attention fell into the quintile of the genes with the most attention (S1 Data). (B) Share of MEDLINE published within indicated year that covers the 5% most-studied genes until the indicated year. For R01, Impact and innovation, Exploratory, and Training grant categories, the share of MEDLINE with support of at least one grant of the respective category is compared against the 5% of genes most studied, irrespective of their grant support. (C) Illustration of the 1,000 genes occurring in the most publications supported by exploratory grants of the NIH in the year 2015. NIH, National Institutes of Health; PI, principal investigator; R01, Research Project Grant. (TIF) [file pbio.2006643.s010.tif]

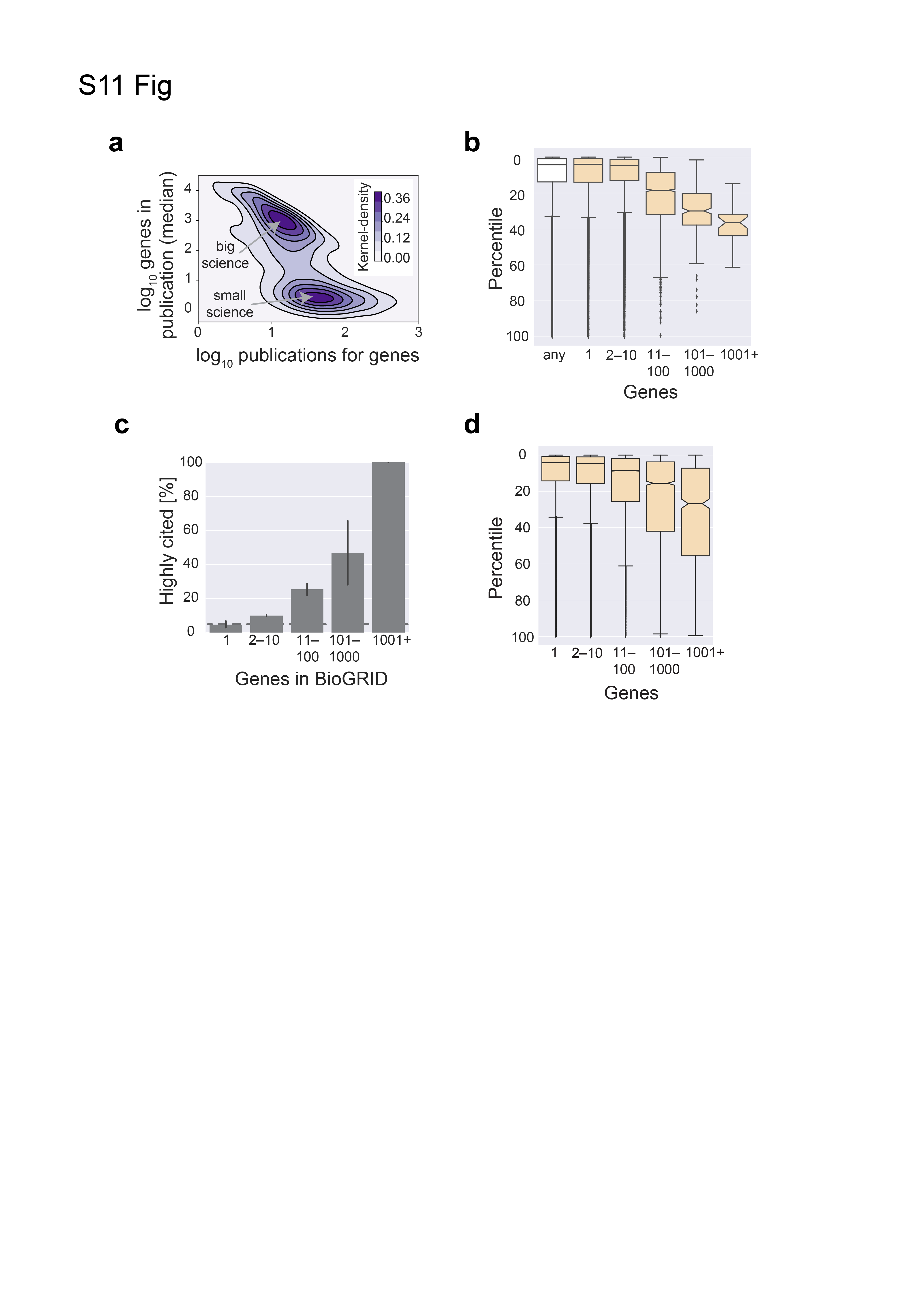

Supplement: S11 Fig — (A) Kernel-density estimation of the fraction of genes with a given number of publications versus the median number of genes co-occurring in the respective publications. The observed pattern is consistent with the notions of “small science” and “big science” (S1 Data). (B) Median percentile of attention for publications as a function of the number of genes associated with the publication (same bins as in Fig 4B). (C) Percentage of highly cited publications (top 5%, shown with dashed line) as a function of the number of genes associated with a publication in BioGRID (rather than by MEDLINE). Error bars show 95% confidence interval. (D) Median percentile of the attention given a single-gene publication as a function of the number of genes associated with the publications it cites. (TIF) [file pbio.2006643.s011.tif]

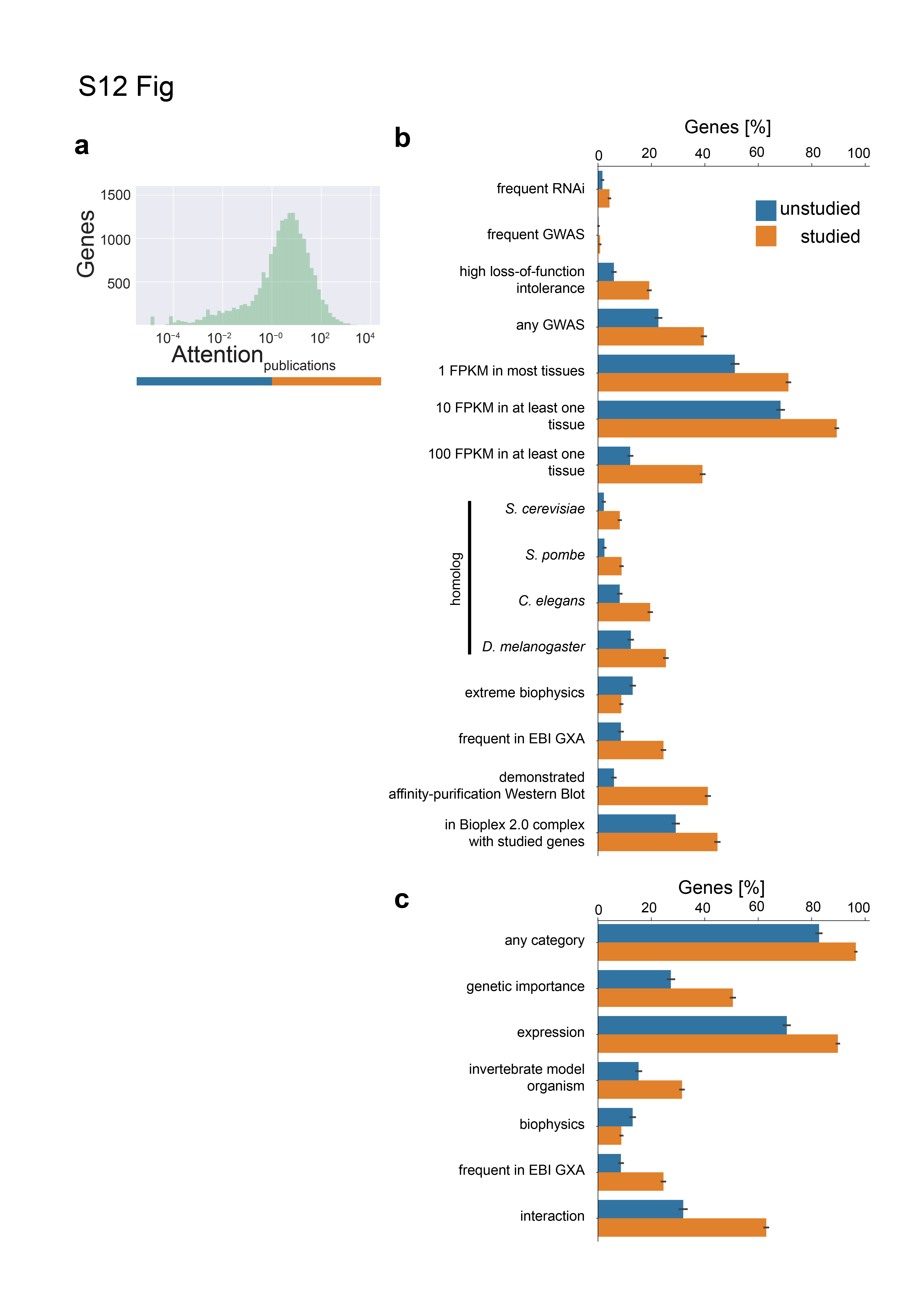

Supplement: S12 Fig — (A) Distribution of the attention (measured by fractional publications) in publications given to genes. Genes with attention levels below 1 are denoted unstudied (blue), whereas genes with attention levels above 1 are denoted studied (orange). (B) Percentage of genes with indicated characteristic. (C) As B, but grouped for the presence of at least one of the characteristics of B. Same order as B. (TIF) [file pbio.2006643.s012.tif]

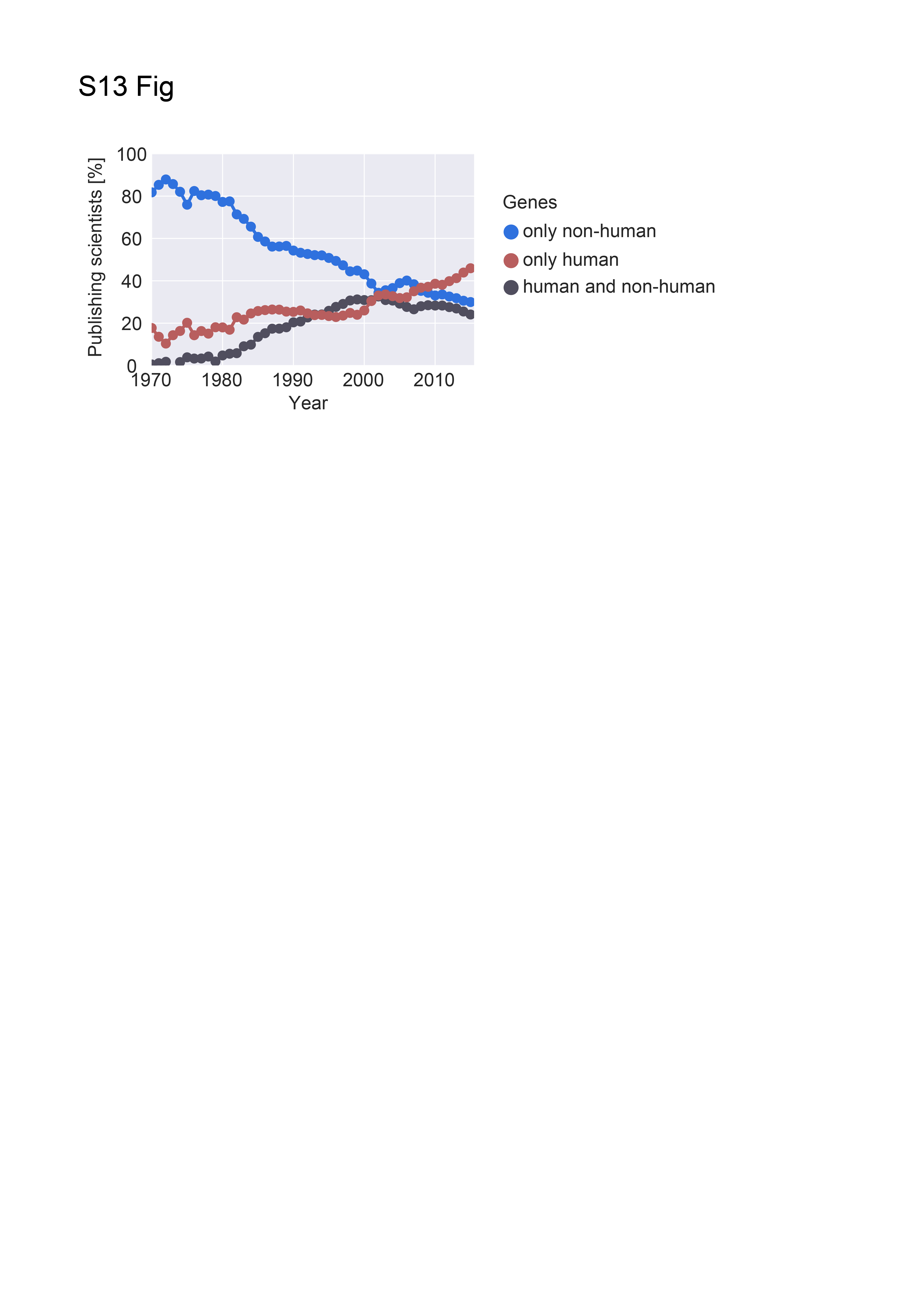

Supplement: S13 Fig — Fraction of scientists who—within the indicated year—publish exclusively on nonhuman genes (or gene products) or exclusively on human genes (or gene products), or both. The fraction of scientists who exclusively published on human genes had been stable in the 1980s and 1990s, while the fraction of scientists working on human and nonhuman genes has been steadily decreasing at the expense of scientists publishing exclusively on nonhuman genes. Around the year 2000, the fraction of scientists working on human and nonhuman genes started to plateau, while the fraction of scientist working exclusively on human genes increased by approximately 10 percent points and has since been steadily increasing (S1 Data). (TIF) [file pbio.2006643.s013.tif]
